# Sonodynamic CoMg-Quercetin Nanozyme for Antibacterial Therapy and Multifunctional Bone Regeneration in Infectious Bone Defects

**DOI:** 10.34133/bmr.0396

**Published:** 2026-07-14

**Authors:** Yurong Xu, Jingyu Yan, Lihong Zhou, Chenying Cui, Kaifang Zhang, Kun Liu, Xiuping Wu, Bing Li

**Affiliations:** ^1^ Shanxi Medical University School and Hospital of Stomatology, Taiyuan 030001, Shanxi, China.; ^2^ Shanxi Province Key Laboratory of Oral Diseases Prevention and New Materials, Taiyuan 030001, Shanxi, China.

## Abstract

Infectious bone defects remain a serious clinical challenge because infection-associated inflammation markedly hinders bone regeneration. To address this complicated pathological condition, a porphyrin-doped cobalt-magnesium bimetallic quercetin-based sonosensitive nanozyme (CoMg-Que) was engineered to integrate antibacterial therapy, redox modulation, and tissue regenerative functions within a single platform. Upon ultrasound (US) irradiation, CoMg-Que was activated and efficiently generated reactive oxygen species (ROS) for sonodynamic antibacterial treatment. Following termination of US stimulation, its inherent superoxide dismutase- and catalase-like activities further removed excessive ROS, thereby relieving inflammatory responses and re-establishing redox homeostasis. In vitro experiments demonstrated that CoMg-Que effectively eliminated methicillin-resistant *Staphylococcus aureus* (MRSA) and exhibited favorable immunomodulatory, pro-angiogenic, and osteogenic properties. In an MRSA-infected rat calvarial defect model, CoMg-Que achieved an antibacterial efficiency of 95.24% and markedly enhanced bone regeneration, with the bone volume fraction reaching 26.76%. These findings indicate that CoMg-Que may serve as a promising multifunctional platform for infectious bone defect treatment through coordinated antibacterial, immunoregulatory, and regenerative activities.

## Introduction

Infectious bone defects (IBDs) represent a major challenge in orthopedic clinical practice and are characterized by a vicious cycle involving persistent infection and impaired bone regeneration [1]. At present, many materials designed for IBD aim to simultaneously achieve antibacterial and anti-inflammatory effects by combining reactive oxygen species (ROS)-mediated bactericidal activity with ROS-scavenging capability [2–4]. Nevertheless, the dynamic balance between these 2 contradictory processes remains difficult to accurately control, often leading to either inadequate bactericidal efficacy or tissue injury induced by excessive oxidative stress. Therefore, developing a therapeutic strategy capable of realizing controllable ROS regulation at different pathological stages is of considerable importance. In recent years, sonodynamic therapy (SDT), as a noninvasive antibacterial approach with deep tissue penetration and spatiotemporal controllability, has attracted widespread interest in infectious disease therapy [5,6]. The bactericidal mechanism mainly relies on the following process: under the coexistence of water and oxygen, the sonosensitizer cooperates with ultrasound (US) to promote ROS generation through electron transfer, thereby producing antibacterial effects [7]. ROS can eradicate pathogenic bacteria by disrupting the electron transport chain, resulting in oxidative stress and dysfunction of energy metabolism. However, ROS also exerts dual biological effects, particularly within the complex inflammatory microenvironment of IBD [8]. While effectively killing bacteria, ROS may simultaneously induce secondary injury to surrounding normal cells and tissues at the infection site. Previous studies have demonstrated that ROS can drive macrophage polarization toward the M1 phenotype, thereby aggravating the local inflammatory response and sustaining inflammation progression [9]. In addition, ROS may induce mitochondrial dysfunction in stem cells, thus interfering with their osteogenic differentiation process and ultimately affecting tissue repair [10]. An ideal therapeutic system should achieve temporally regulated ROS modulation: generating ROS during the early infection stage to realize rapid antibacterial activity, while promptly scavenging excessive ROS during the later stage to alleviate inflammation and facilitate tissue repair. However, most currently reported SDT nanoplatforms have predominantly concentrated on antibacterial performance [11,12], while largely overlooking the secondary damage to the immune and osteogenic microenvironment caused by residual ROS after SDT treatment [13], which greatly restricts their therapeutic potential in complex infectious bone repair.

To mitigate the detrimental effects of ROS, commonly employed free radical scavenging strategies mainly include inorganic nanomaterials, anti-inflammatory gas therapies based on nitric oxide or carbon monoxide, and naturally derived active compounds [14]. Among these candidates, natural polyphenolic compounds exhibit unique advantages because of their excellent biocompatibility, inherent anti-inflammatory and antioxidant properties, and favorable biosafety profiles [15]. As a representative flavonoid polyphenol, quercetin (Que) has been extensively verified to possess diverse biological functions, including ROS-scavenging, anti-inflammatory, and bone repair-promoting activities [16]. These biological effects are mainly associated with inhibition of inflammatory signaling pathways such as NF-κB and MAPK, thereby decreasing the secretion of pro-inflammatory cytokines, including TNF-α, IL-6, and IL-1β, while simultaneously attenuating oxidative stress responses [17]. Nevertheless, the practical biomedical application of Que remains greatly restricted because of poor water solubility, limited stability, and low in vivo bioavailability. To overcome these limitations, metal-polyphenol coordination engineering has emerged as an effective approach [18]. Through coordination interactions between metal ions and catechol/phenolic hydroxyl groups within polyphenol molecules, the stability and bioavailability of natural polyphenols can be substantially improved while simultaneously conferring multifunctional biological properties to the system [19]. More importantly, by rationally incorporating metal ions with distinct biological functions, multimetal synergistic composite systems can be established. Such a strategy enables integration of multiple biological properties—including antibacterial, antioxidant, anti-inflammatory, and osteogenic activities—at the molecular level, thereby achieving functional synergy and intelligent regulation that are difficult to realize using single-component systems [20,21]. However, current metal-polyphenol platforms have mainly been explored in traditional bone repair or drug delivery studies [22–24], whereas studies truly integrating US-enhanced antibacterial activity, ROS-responsive immune modulation, and osteogenic repair within a single metal-polyphenol nanosystem remain very limited. In particular, there is still a lack of therapeutic systems capable of achieving temporally coordinated “antibacterial–anti-inflammatory–regeneration” therapy within the complicated IBD microenvironment.

**Fig. 1.**
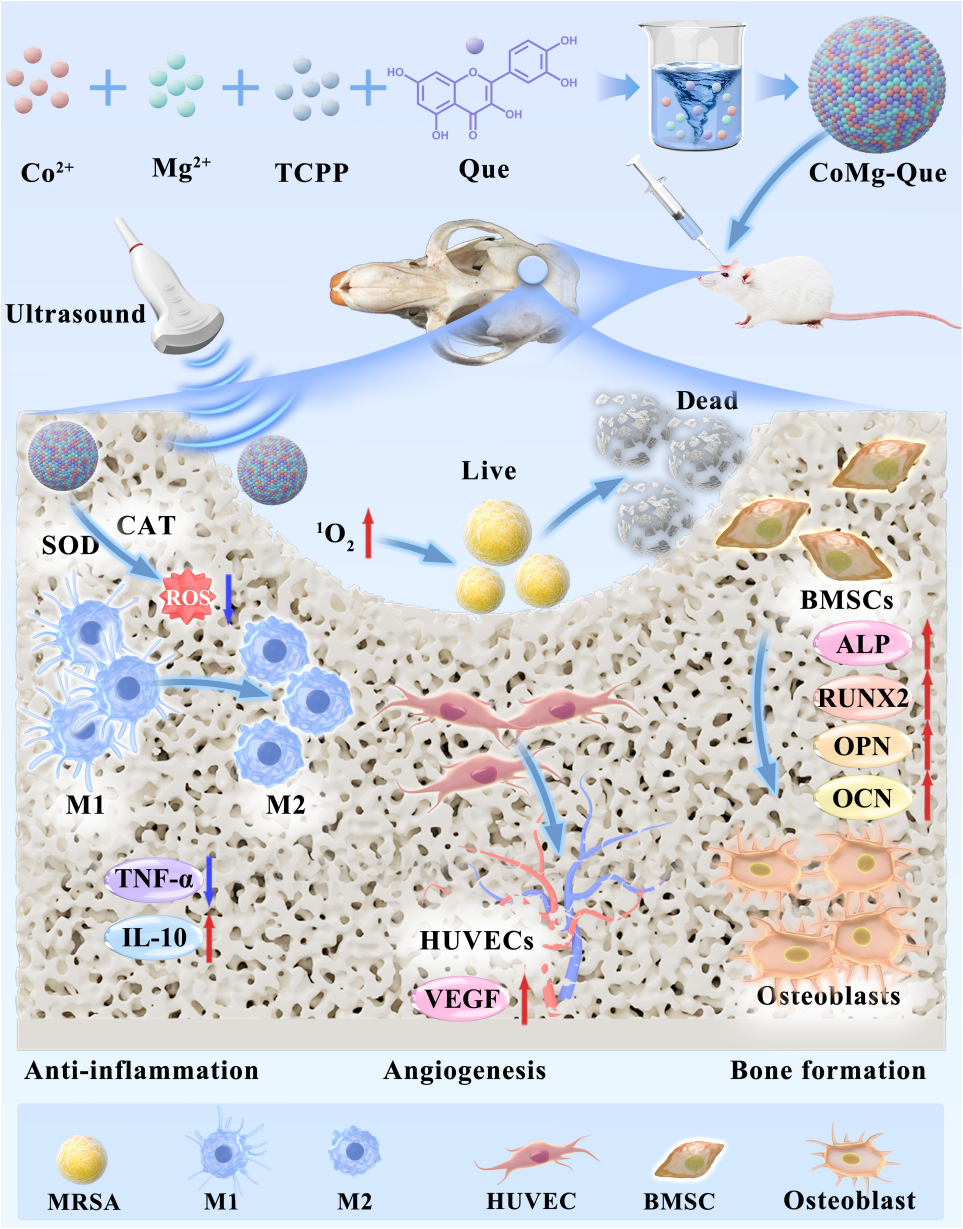
Synthesis of CoMg-Que and its multifunctional role in infected bone defects.

In the present study, a porphyrin-doped cobalt-magnesium bimetallic quercetin-based sonodynamic nanoplatform (CoMg-Que) was fabricated through a coordination-driven self-assembly strategy (Fig. 1). The design concept of this platform was based on the stage-dependent pathological characteristics of IBD, in which rapid antibacterial action is required during the early infection stage, whereas inflammation modulation and bone tissue regeneration are necessary during the later stage. Accordingly, Co^2+^, Mg^2+^, and Que were functionally integrated to achieve temporally synergistic “antibacterial–anti-inflammatory–regeneration” therapy. Specifically, Co^2+^ mainly functions as the catalytic center for regulating the infectious microenvironment. Because of its catalase (CAT)-like activity [25], Co^2+^ can effectively catalyze the decomposition of endogenous H_2_O_2_ to produce oxygen, thereby alleviating local hypoxia and supplying sufficient oxygen for SDT. This characteristic overcomes the hypoxic limitation commonly observed in infected tissues and produces a “self-enhancing” antibacterial effect. Meanwhile, Co^2+^ also exhibits superoxide dismutase (SOD)-like activity [26], allowing further scavenging of excessive ROS after SDT treatment and thereby reducing oxidative stress-induced inflammatory injury. In addition, previous investigations have demonstrated that Co^2+^ can promote angiogenesis, which further contributes to the reconstruction of the regenerative microenvironment [27]. In contrast, Mg^2+^ is mainly involved in the later-stage tissue regeneration process. As an essential trace element with excellent biocompatibility and degradability, Mg^2+^ participates in various enzymatic reactions and has been widely recognized as a classical osteogenic metal ion. Increasing evidence has confirmed that Mg^2+^ can directly enhance osteoblast proliferation, osteogenic differentiation, and extracellular matrix mineralization, thereby facilitating bone tissue regeneration [28]. More importantly, Que not only possesses intrinsic antioxidant and anti-inflammatory properties but also acts as a multifunctional coordination ligand through its abundant phenolic hydroxyl groups, enabling stable incorporation of Co^2+^ and Mg^2+^ into the same nanosystem. This coordination-driven nanostructure not only improves the stability and bioavailability of Que but also enables synergistic integration of multiple functions—including SDT-enhanced antibacterial activity, ROS regulation, pro-angiogenic effects, and osteogenic promotion—within a unified platform.

Based on the synergistic mechanisms described above, CoMg-Que is expected to exert temporally regulated therapeutic functions within the IBD microenvironment: under ultrasonic stimulation, the system rapidly produces ROS to efficiently eradicate pathogenic bacteria; after completion of SDT, its CAT/SOD-like enzymatic activities further remove excessive ROS, thereby alleviating oxidative stress and suppressing inflammatory responses; meanwhile, the sustained release of Co^2+^, Mg^2+^, and Que synergistically promotes angiogenesis and osteogenic differentiation, ultimately accelerating bone tissue regeneration. Overall, this multifunctional nanosystem provides a promising strategy for constructing a closed-loop therapeutic paradigm integrating “antibacterial–anti-inflammatory–bone regeneration.”

## Materials and Methods

### Chemicals

*N*,*N*-dimethylformamide (DMF, ≥99.8%), tetrakis (4-carboxyphenyl) porphyrin (TCPP, ≥97%), anhydrous cobalt(II) chloride (CoCl_2_, ≥99.5%), anhydrous magnesium chloride (MgCl_2_, ≥99.5%), and Que (≥98%) were obtained from Aladdin (China).

### Preparation of Co-Que and CoMg-Que

CoMg-Que: MgCl_2_ (1.0 mmol), CoCl_2_ (1.0 mmol), Que (0.5 mmol), and TCPP (0.05 mmol) were dissolved in 30 ml of DMF. The molar ratio of 1.0:1.0:0.5:0.05 was established through systematic optimization: equimolar Mg^2+^ and Co^2+^ promote bimetallic electronic synergy, while the 1:4 ratio between Que and total metal ions ensures adequate chelation without aggregation of free Que. TCPP was introduced as a minor modulating component to facilitate homogeneous nucleation. Through screening of different compositional ratios, this formulation exhibited the most favorable product consistency, yield, and morphology. The reaction mixture was magnetically stirred at room temperature for 6 h and then centrifuged (12,000 rpm, 20 min, 4 °C) to collect the dark brown colloidal product. The obtained precipitate was sequentially rinsed with hot DMF (2 × 20 ml, 80 °C), ethanol (2 × 20 ml), and ultrapure water (3 × 20 ml). After freeze-drying (–50 °C, 0.05 mbar, 48 h), the purified CoMg-Que nanomaterial was obtained as a fluffy dark brown powder and preserved in a desiccator before further use. The synthesis procedure was independently repeated 3 times, yielding 78.93%, 77.45%, and 79.26%, respectively (average 78.55% ± 0.91%), indicating good reproducibility with minimal batch variation.

Co-Que: CoCl_2_ (2.0 mmol), Que (0.5 mmol), and TCPP (0.05 mmol) were dissolved in 30 ml of DMF, and the following procedures were performed identically to those described for CoMg-Que. For in vitro studies, the freeze-dried powders were prepared as stock suspensions with defined concentrations. Briefly, Co-Que or CoMg-Que powders were accurately weighed and dispersed in an appropriate solvent, followed by sonication in an ice-water bath for 10 to 30 min to obtain a uniform stock suspension at 1 mg/ml. Subsequently, working suspensions with the required concentrations (e.g., 50, 100, 150, 200, and 250 μg/ml) were freshly prepared by diluting the stock suspension with the same solvent according to experimental requirements.

### Characterization

Morphological characteristics were observed using scanning electron microscopy (SEM), while the nanostructure was further characterized by transmission electron microscopy (TEM). The hydrodynamic diameter and zeta potential were measured by dynamic light scattering (DLS) to assess size distribution and surface charge, respectively. Elemental composition and chemical valence states were analyzed using x-ray photoelectron spectroscopy (XPS). Functional groups were identified by Fourier transform infrared spectroscopy (FT-IR), and crystalline structures were examined through x-ray diffraction (XRD).

### Free radical and ROS scavenging ability

First, the total antioxidant activity was evaluated using the ABTS (Sigma-Aldrich, USA) radical scavenging assay by measuring inhibition of the characteristic absorbance of ABTS•^+^ at 734 nm (or 405 nm) after sample treatment. This assay was further supplemented by the DPPH (Sigma-Aldrich, USA) radical scavenging test, in which the reduction in absorbance of the DPPH solution at 515 nm was monitored to calculate the scavenging efficiency. In addition, SOD-like activity was determined using the NADH-mPMS-NBT (Sigma-Aldrich, USA) colorimetric method to evaluate the scavenging capacity toward superoxide anion radicals (•O_2_^–^), whereas CAT-like activity was assessed through a dissolved oxygen assay to determine the catalytic efficiency for hydrogen peroxide decomposition. All experiments described above were conducted using 50 μg/ml of the materials at room temperature.

### Ion release

To investigate ion release behavior, the specimens were immersed in simulated body fluid (SBF, AR; Aladdin, China) adjusted to pH 6.0 and 7.4 (HCl/NaOH, AR; Sinopharm, China), with continuous shaking at 37 °C in a water bath shaker. At predetermined time points, aliquots of the immersion solution were collected from centrifuge tubes for ionic analysis and immediately replenished with an equal volume of fresh SBF to maintain sink conditions. The cumulative release profiles of Co^2+^ and Mg^2+^ were quantitatively analyzed by inductively coupled plasma optical emission spectrometry (ICP-OES) at each sampling interval.

### US responsiveness test

Singlet oxygen (^1^O_2_) generation after 10 min of US irradiation was detected by electron spin resonance (ESR) spectroscopy, using 2,2,6,6-tetramethylpiperidine (TEMP, ≥98%; Aladdin, China) as the spin-trapping agent. In addition, ^1^O_2_ production was further evaluated using DPBF (Sigma-Aldrich, USA) as an indicator probe. Briefly, 250 μl of a mixed solution containing 50 μg/ml materials and 50 μM DPBF was transferred into a 96-well plate and exposed to ultrasonic irradiation in the dark for 10 min. The absorbance at 420 nm was recorded every 2 min. The pseudo-first-order reaction rate constant was calculated according to ln(*A*_t_/*A*_0_) = –*kt*, where *A*_0_ and *A*_t_ represent absorbance values of DPBF at the start and after a specific time *t*, respectively, measured at 420 nm. The rate constant *k* was determined by fitting the data to a linear function. US irradiation was conducted at 1.0 MHz, 1.5 W/cm^2^, and a 50% duty cycle for 10 min, according to previously reported conditions capable of efficiently generating ^1^O_2_ while minimizing heating and sample degradation [29,30]. All US-related experiments in this work were performed under the same irradiation parameters.

### CCK-8 assay

To assess the cytotoxicity of the materials toward human umbilical vein endothelial cells (HUVECs, ATCC, USA), murine macrophages (RAW264.7, ATCC, USA), and bone marrow mesenchymal stem cells (BMSCs, ATCC, USA), cell viability was determined using a CCK-8 assay. HUVECs were seeded into 96-well plates at a density of 5 × 10^4^ cells per well and allowed to adhere overnight. Subsequently, the cells were exposed to materials at concentrations ranging from 50 to 250 μg/ml for 5 days. After treatment, the wells were washed with phosphate-buffered saline (PBS), and 100 μl of medium containing 10% CCK-8 reagent (Dojindo, Japan) was added, followed by incubation in the dark. The same experimental procedure was similarly applied to the other cell types.

### Hemolysis assay

A 1-ml aliquot of 5% rat RBC suspension was centrifuged at 200 ×*g* for 15 min, washed 3 times with saline, and then resuspended. CoMg-Que was added to achieve final concentrations ranging from 50 to 250 μg/ml, while distilled water and saline (0.9% NaCl; Sinopharm, China) were used as positive and negative controls, respectively. Following incubation at 37 °C for 1 h, the samples were centrifuged again at 200 ×*g* for 15 min, and 100 μl of the supernatant was collected for absorbance measurement at 540 nm.

### Live/dead assay

HUVECs and RAW264.7 cells (1 × 10^4^ cells per well) were inoculated into 24-well plates and cultured overnight for cell attachment. For the Co-Que+US and CoMg-Que+US groups, 100 μl of material suspension (200 μg/ml) was introduced, followed by US irradiation (1.0 MHz, 1.5 W/cm^2^, 50% duty cycle) for 10 min. After an additional 24 h of incubation, the cells were rinsed with PBS and stained using a live/dead staining kit (Solarbio, China) for 15 min. Excess dye solution was removed, and cell viability was observed under a confocal laser scanning microscope (CLSM).

### Antibacterial performance

The antibacterial and anti-biofilm activities of CoMg-Que were systematically evaluated through multiple assays. For preliminary antibacterial screening, *Escherichia coli* (*E. coli*, ATCC 25922, USA) and *Staphylococcus aureus* (*S. aureus*, ATCC 29213, USA) (1 × 10^8^ CFU/ml) were spread onto agar plates (Solarbio, China), and 6-mm discs loaded with different concentrations of CoMg-Que were placed on the surface. After incubation at 37 °C for 24 h, inhibition zones were measured to evaluate concentration-dependent antibacterial activity under non-US conditions. To investigate sonodynamic antibacterial effects, *E. coli*, *S. aureus*, and methicillin-resistant *Staphylococcus aureus* (MRSA, ATCC 43300, USA) (10^7^ CFU/ml, diluted 200-fold with 200 μg/ml materials) were cultured at 37 °C for 24 h with or without US irradiation (1.0 MHz, 1.5 W/cm^2^, 50% duty cycle, 10 min). Bacterial viability was subsequently evaluated by colony counting and live/dead staining (SYTO9/PI, Solarbio, China). For morphological observation, bacteria were harvested, rinsed with PBS, fixed using 2.5% glutaraldehyde (Sinopharm, China) at 4 °C overnight, dehydrated through graded ethanol solutions (75%, 85%, 95%, and 100%; Sinopharm, China), sputter-coated with gold, and observed using SEM. Biofilm formation was quantitatively assessed by crystal violet staining. Briefly, bacteria (1 × 10^8^ CFU/ml) were cultured in 24-well plates for 24 h, followed by treatment with different materials and, for the US groups, additional US irradiation. After washing, the biofilms were stained with 0.1% crystal violet (Sigma-Aldrich, USA) for 15 min and dissolved in ethanol, and the absorbance at 570 nm was measured to evaluate biofilm disruption.

### Intracellular ROS scavenging capacity

The intracellular ROS-scavenging capability was examined using the fluorescent probe 2′,7′-dichlorodihydrofluorescein diacetate (DCFH-DA, ≥97%; Sigma-Aldrich, USA). HUVECs and RAW264.7 cells were seeded into 24-well plates at a density of 2 × 10^4^ cells per well. After treatment with 50 μM H_2_O_2_ and different materials (200 μg/ml) for 24 h, the cells were washed with PBS and incubated with DCFH-DA for 30 min. Subsequently, fresh culture medium was used to rinse the cells, and fluorescence signals were visualized using a fluorescence microscope to reflect intracellular ROS levels.

### Immunofluorescence staining

HUVECs, RAW264.7 cells, and BMSCs were individually cocultured with materials (200 μg/ml) from different experimental groups at a density of 4 × 10^5^ cells/ml for 48 h, while LPS (from *E. coli* O111:B4, Sigma-Aldrich, USA) was added into the RAW264.7 culture system for inflammatory stimulation. After incubation, the cells were fixed with 4% paraformaldehyde (Servicebio, China) at room temperature for 30 min, followed by 2 PBS washes and permeabilization with 0.1% Triton X-100 (Sigma-Aldrich, USA) for 15 min. Subsequently, the cells were blocked with blocking solution (5% BSA, Solarbio, China) for 60 min to minimize nonspecific binding. Primary antibodies were then added and incubated overnight at 4 °C as follows: HUVECs were treated with anti-VEGF (Affinity Biosciences, USA); RAW264.7 cells were incubated with anti-CD86, anti-iNOS, and anti-CD206 (Affinity Biosciences, USA); and BMSCs were treated with anti-RUNX2 and anti-OCN (Affinity Biosciences, USA). Subsequently, cells were incubated with species-matched secondary antibodies (Alexa Fluor 488, Abcam, UK) for 1 h at room temperature in the dark. After PBS washing, the cytoskeleton was stained using phalloidin (Abcam, UK) for 30 min, followed by nuclear counterstaining with 4′,6-diamidino-2-phenylindole (DAPI; Sigma-Aldrich, USA) for 5 min. Finally, all samples were visualized using CLSM.

### Cell scratch, migration, and tube formation assays

The influence of the materials on HUVEC migration and angiogenesis was investigated using wound healing, Transwell migration, and Matrigel tube formation assays. For the wound healing assay, HUVECs (5 × 10^4^ cells/well) were seeded into 12-well plates, cultured overnight, and scratched using a sterile pipette tip. Cells were then treated with different materials (200 μg/ml), and images were collected at 0 and 24 h. The wound area was quantified using ImageJ, and the healing rate (%) was calculated according to [(*A*_0_ – *A*_t_)/*A*_0_] × 100%, where *A*_0_ and *A*_t_ represent scratch areas at 0 and 24 h, respectively. For the Transwell migration assay, HUVECs (1 × 10^5^ cells/well) were seeded into the upper chambers, whereas the lower chambers contained medium supplemented with materials (200 μg/ml). After 24 h, migrated cells on the lower membrane surface were fixed with 4% paraformaldehyde (Servicebio, China), stained using 0.1% crystal violet (Sigma-Aldrich, USA), and counted. For angiogenesis analysis, 96-well plates were coated with 50 μl of Matrigel (Corning, USA) per well and allowed to polymerize. HUVECs treated with different materials (200 μg/ml) were seeded onto the Matrigel layer and cultured for 6 h. Capillary-like structures were imaged from 3 random fields in each well, and the numbers of nodes and segments were quantified using ImageJ.

### Alkaline phosphatase staining

Alkaline phosphatase (ALP) activity in BMSCs was analyzed using a BCIP/NBT kit (Solarbio, China). After 14 days of culture with different materials (200 μg/ml) in osteogenic medium (Gibco, USA), the cells were fixed with 4% paraformaldehyde (Servicebio, China) for 30 min and stained with BCIP/NBT solution in the dark for 1 h. Excess staining solution was removed by PBS washing, and ALP-stained cells were observed using an optical microscope.

### Alizarin Red S staining

After 21 days of coculture with different materials (200 μg/ml), BMSCs were fixed using 4% paraformaldehyde (Servicebio, China) for 15 min, followed by staining with 0.1% Alizarin Red S (ARS) (Solarbio, China) at 37 °C for 30 min. Following staining, the cells were thoroughly washed with PBS, and mineralized nodules were visualized and imaged under an optical microscope.

### Quantitative real-time PCR

Total RNA was isolated using an RNA extraction kit (Vazyme, China) and reverse-transcribed into cDNA using the PrimeScript RT kit (Vazyme, China). Quantitative real-time PCR (qRT-PCR) was conducted on a QuantStudio 6 Flex system (Thermo Fisher Scientific) using SYBR Premix Ex Taq (Takara, Japan). GAPDH served as the internal reference gene, and relative gene expression levels were calculated using the 2^–ΔΔCt^ method. Primer sequences are provided in Table S1.

### Transcriptome sequencing

To explore the molecular mechanisms responsible for these biological effects, BMSCs were cocultured with CoMg-Que (200 μg/ml) and induced toward osteogenic differentiation for 14 days. Total RNA was isolated using TRIzol (Invitrogen, USA), and sequencing libraries were constructed for analysis on the BGISEQ-500 platform (BGI, Shenzhen, China). Differentially expressed genes (DEGs) were identified using the criteria of fold change > 1.5 or < –1.5 and *P* < 0.05. Functional annotation was conducted using Gene Ontology (GO), and additional bioinformatics analyses were performed through the Bio-Cloud platform.

### Evaluation of in vivo healing of infected bone defects

All animal experiments were approved by the Animal Ethics Committee of the Medical Center of Shanxi Medical University (Approval No. KQDW-2024-004) and conducted in accordance with the Guide for the Care and Use of Laboratory Animals. Twelve healthy male SD rats (≈300 g) were randomly assigned to 4 groups (*n* = 3 per group), with the experimental design following the 3R principles (Replacement, Reduction, and Refinement) and ethical guidelines to minimize animal use while ensuring experimental validity, to establish a 5-mm critical-sized calvarial defect model. Rats were anesthetized by intraperitoneal injection of 2% pentobarbital sodium (2 ml/kg; Sigma-Aldrich, USA), and a 5-mm defect was generated in the right calvarium. Subsequently, 50 μl of MRSA suspension (10^7^ CFU/ml) was introduced into the defect to establish localized infection. Three days later, the infected site was reopened, and sterile PBS, Co-Que solution, or CoMg-Que solution was applied to the defects in the MRSA, Co-Que+US, and CoMg-Que+US groups, respectively. The latter 2 groups were then exposed to US irradiation (1.0 MHz, 1.5 W/cm^2^, 50% duty cycle, 10 min), whereas rats in the Van group received intravenous vancomycin (40 mg/kg; ≥98%; Meilunbio, China) (40 mg/kg). After 8 weeks, the animals were sacrificed, and calvarial specimens were collected for micro-CT scanning, hematoxylin and eosin (H&E) staining, Masson’s trichrome staining, immunohistochemistry, and immunofluorescence analyses. All outcome evaluations were conducted by investigators blinded to the treatment groups. In addition, major visceral organs (heart, liver, spleen, lungs, and kidneys) were harvested for H&E staining to examine potential systemic toxicological manifestations.

### Statistical analysis

Statistical analysis was conducted using GraphPad Prism. Data normality was first examined using the Shapiro–Wilk test. For normally distributed data, differences between 2 groups were analyzed using an unpaired Student’s *t*-test, whereas comparisons among multiple groups were performed using one-way ANOVA followed by Tukey’s post hoc test for multiple-comparison correction. All experiments were performed in triplicate (*n* = 3).

Data are expressed as mean ± standard deviation (SD). Statistical significance was indicated as follows: ns represents *P* > 0.05, *P < 0.05, **P < 0.01, ***P < 0.001, and ****P < 0.0001.

## Results and Discussion

### Structural and morphological characterization

**Fig. 2.**
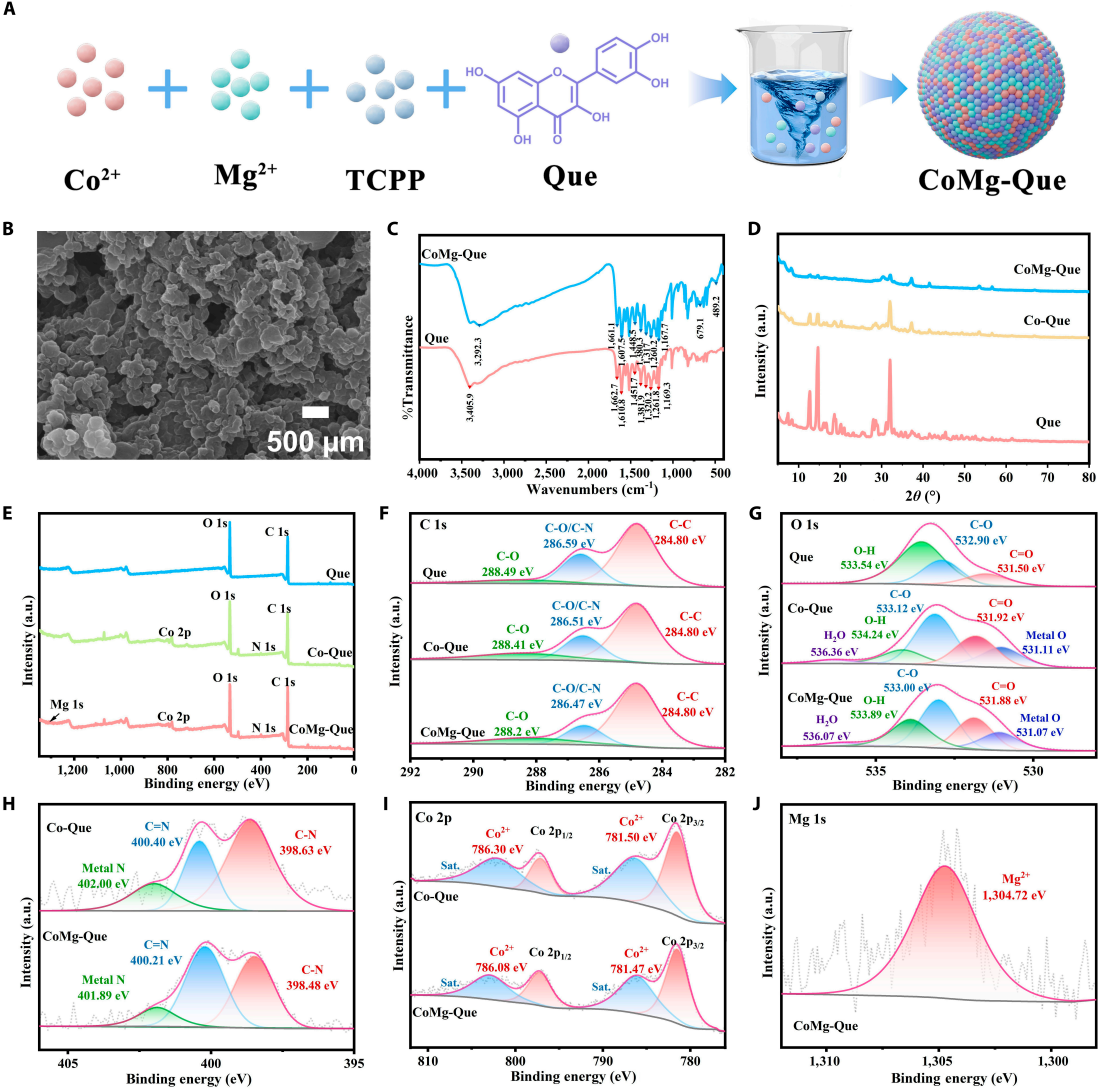
Characterization of CoMg-Que. (A) Schematic diagram illustrating the synthesis of CoMg-Que. (B) SEM image of CoMg-Que. (C) FT-IR spectra. (D) XRD patterns. (E) XPS survey spectra. (F to J) XPS high-resolution scans of C1s, O1s, N1s, Co 2p, and Mg1s.

Using DMF as the solvent, CoMg-Que nanomaterials were prepared at room temperature through coordination-driven self-assembly using TCPP, CoCl_2_, MgCl_2_, and Que as precursor materials (Fig. 2A). First, the morphology was examined by SEM (Fig. 2B), and the results indicated that the material consisted of aggregated near-spherical nanoparticles, forming a relatively uniform 3-dimensional porous network. This morphology is mainly ascribed to the stable coordination between the catechol groups of Que molecules and Co^2+^ and Mg^2+^, which promotes the oriented assembly of nanoparticles. The particle size distribution was further quantified based on SEM image analysis (Fig. S1), revealing a relatively uniform size distribution with an average diameter of 155.89 ± 3.98 nm. DLS further measured an average hydrodynamic diameter of 182.43 nm and a zeta potential of −33.02 mV (Figs. S2 and S3). The slightly larger hydrodynamic size compared to the SEM result is attributed to the hydration layer surrounding the nanoparticles in solution, while the highly negative zeta potential ensures electrostatic repulsion and prevents aggregation. The physicochemical characteristics of CoMg-Que were systematically summarized in Table S2. To evaluate batch-to-batch reproducibility, 3 independent batches were prepared under identical conditions and characterized by SEM and DLS. The results demonstrated highly consistent core sizes (155.89 ± 5.71 nm), hydrodynamic diameters (182.43 ± 3.23 nm), and zeta potentials (−33.02 ± 2.12 mV) across all batches, indicating minimal batch-to-batch variation and confirming the robustness of the synthesis protocol. To further investigate the microstructure, the material was analyzed using TEM. TEM images showed that the morphology of CoMg-Que was comparable to that of Co-Que (Fig. S4). Subsequently, FT-IR spectroscopy was carried out to characterize CoMg-Que (Fig. 2C). Compared with pure Que, the O–H stretching vibration peak (from 3,405.9 to 3,292.3 cm^–1^) and the C=O stretching vibration peak (from 1,662.7 to 1,661.1 cm^–1^) in CoMg-Que both shifted toward lower wavenumbers. This red shift is mainly attributed to disruption of intermolecular/intramolecular hydrogen bonds and coordination of carbonyl and hydroxyl groups with metal ions. Moreover, the positions of characteristic peaks related to the aromatic ring were also distinctly changed. Notably, 2 new peaks appeared in CoMg-Que at 679.1 and 489.2 cm^–1^, which were assigned to the stretching vibrations of Co–O and Mg–O bonds, confirming the formation of metal-oxygen coordination bonds. These results indicate that metal ions were successfully coordinated with Que. XRD analysis further showed that metal ion coordination markedly decreased the crystallinity of Que, with CoMg-Que displaying an amorphous structure (Fig. 2D).

After characterization of the crystal structure and chemical bonding states of the material, XPS analysis was conducted on Co-Que and CoMg-Que to further clarify the surface elemental chemical states and coordination environment. Survey spectra (Fig. 2E) detected C, O, N, and Co in Co-Que, with additional Mg detected in CoMg-Que, confirming the formation of the bimetallic system. High-resolution spectra (Fig. 2F to J) provided distinct peak assignments: in the C 1s spectrum, the C–O peak shifted from 288.49 to 288.20 eV, suggesting electron donation from oxygen atoms to metal ions. In the O 1s spectrum, a new Metal–O peak appeared at 531.11 eV (Co-Que) and 531.07 eV (CoMg-Que), directly demonstrating coordination between the carbonyl and hydroxyl groups of Que and the metal ions. In the N 1s spectrum, a Metal–N peak appeared at 402.00 eV (Co-Que) and 401.89 eV (CoMg-Que), indicating that nitrogen atoms in TCPP were also involved in coordination. The slight changes in the binding energy of Co 2p revealed the regulatory effect of Mg^2+^ introduction on the electronic structure of Co. These findings support the red shift of characteristic peaks and the emergence of new M–O bond vibration peaks observed in FT-IR, as well as the amorphous coordination polymer structure shown by XRD. Collectively, these results confirm the successful construction of a sonosensitive bimetallic polyphenol nanomaterial, in which Co^2+^, Mg^2+^, TCPP, and Que were integrated through multiple coordination bonds.

Before proceeding to biological studies, it is crucial to assess the colloidal stability of the nanozyme under physiological conditions. Thus, the hydrodynamic diameter and zeta potential of the nanoparticles were monitored in PBS and DMEM (10% FBS) for 14 days (Fig. S5). No evident aggregation or marked variation in particle size and surface charge was detected during the incubation period, indicating that CoMg-Que retained good dispersion stability in physiological media. Given that CoMg-Que was designed for SDT, its structural stability under the applied US conditions was further examined by comparing FTIR spectra before and after US exposure (Fig. S6). The main characteristic peaks showed no evident changes after US treatment, indicating that the coordination framework of CoMg-Que remained structurally stable. Collectively, these stability data confirm that the nanoplatform possesses favorable physicochemical properties for subsequent biomedical applications.

### Antioxidant properties

**Fig. 3.**
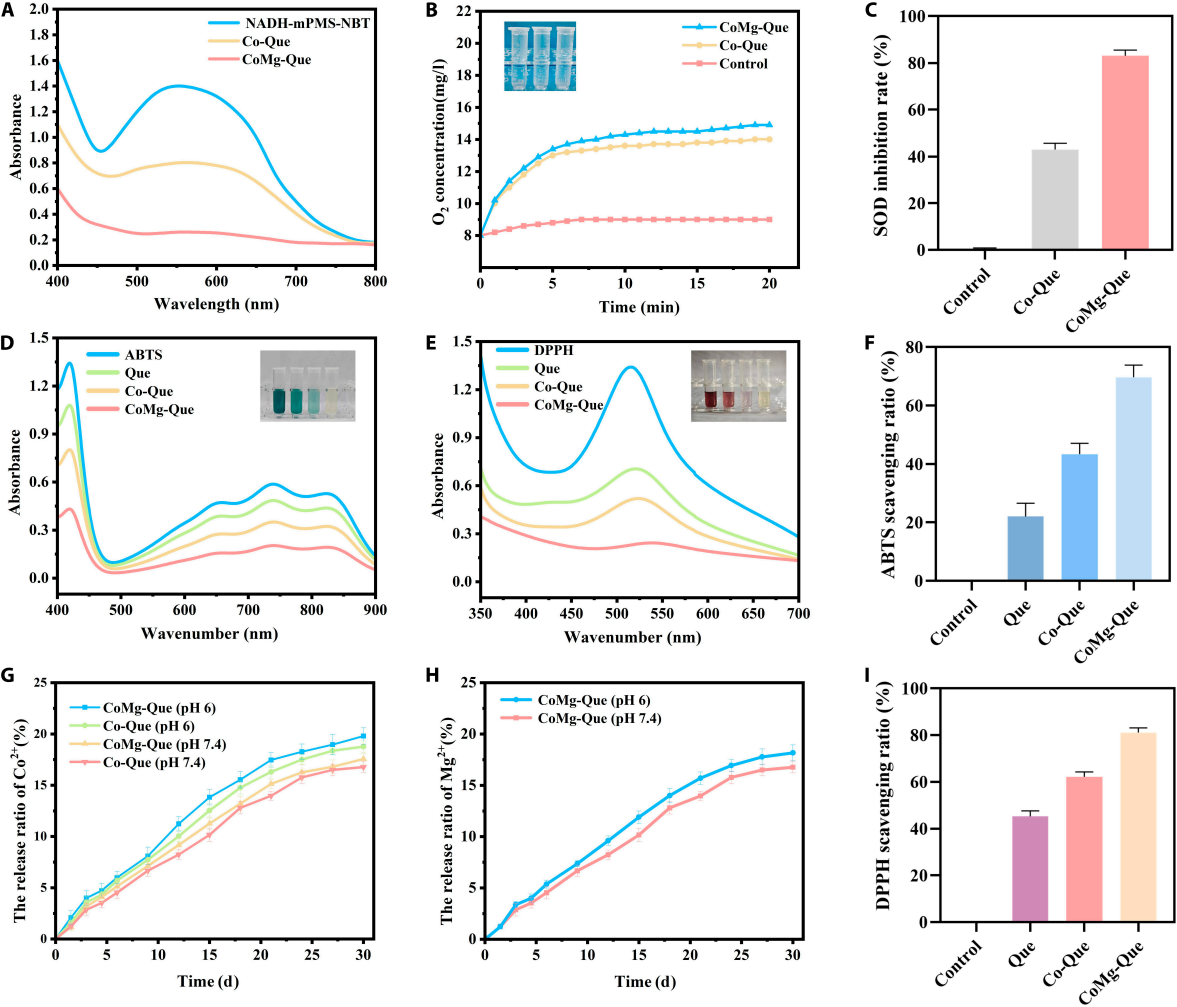
Antioxidant activity. (A) NADH-PMS-NBT assay for evaluating SOD-like activity of different materials (50 μg/ml). (B) Oxygen generation ability of different materials (50 μg/ml) from H_2_O_2_ decomposition over 20 min. (C) Comparison of SOD-like activities among different groups. (D) Absorbance variation of ABTS•^+^ at 734 nm recorded for different material groups (50 μg/ml). (E) Absorbance variation of DPPH radicals at 515 nm recorded for different material groups (50 μg/ml). (F) ABTS radical scavenging percentages. (G and H) Cumulative release profiles of Co^2+^ and Mg^2+^ from Co-Que and CoMg-Que (50 μg/ml) over 30 days in SBF (pH 6.0 and 7.4, 37 °C). (I) DPPH radical scavenging rate. Data are expressed as mean ± SD (*n* = 3).

Excessive ROS accumulation within the pathological microenvironment of infected bone defects causes severe cellular injury, thereby fundamentally impairing the bone healing process [31]. Therefore, efficient removal of excessive ROS is an essential prerequisite for restoring tissue homeostasis and promoting effective bone repair. Intracellular redox balance is regulated through the cascade synergy of multiple antioxidant enzymes, among which the classical SOD/CAT cascade pathway serves as the core enzymatic system responsible for ROS elimination [32]. Based on this concept, the NADH-mPMS-NBT system was first employed to compare the SOD-like activities of Co-Que and CoMg-Que (Fig. 3A). In this system, NADH reacts with mPMS to generate •O_2_^–^, which subsequently oxidizes NBT and produces a characteristic absorption peak at 560 nm. The results demonstrated that, after the addition of CoMg-Que, the absorbance at 560 nm markedly decreased, while the SOD inhibition rate increased from 42.96% to 83.05% (Fig. 3C). This enhancement was more obvious than that observed for Co-Que, indicating that CoMg-Que possesses superior •O_2_^–^ scavenging capability. Furthermore, the CAT-like activities of the materials were investigated through dissolved oxygen monitoring experiments (Fig. 3B). CoMg-Que and Co-Que were separately introduced into buffer solutions containing hydrogen peroxide, where the materials catalyzed H_2_O_2_ decomposition to generate oxygen. Dissolved oxygen measurements revealed that both CoMg-Que and Co-Que exhibited strong CAT-like activity; however, no statistically significant difference in dissolved oxygen increase was observed, suggesting that incorporation of Mg^2+^ did not substantially influence the CAT-like catalytic performance of the material. The antioxidant capability of CoMg-Que was further evaluated using the ABTS radical scavenging assay. As shown in Fig. 3D, ABTS is oxidized into ABTS•^+^, producing a blue-green solution with characteristic absorption peaks at 405 and 734 nm. Different materials reduced ABTS•^+^, resulting in lighter solution color and decreased absorbance intensity. CoMg-Que exhibited strong antioxidant performance, achieving an ABTS•^+^ scavenging efficiency of 69.66% (Fig. 3F). In addition, DPPH dissolved in ethanol presents a purple color with a characteristic absorption peak at 517 nm. After treatment with different materials, the absorption peak declined and the solution exhibited obvious decolorization (Fig. 3E). The results demonstrated that, at identical concentrations, the DPPH scavenging capability followed the order of CoMg-Que > Co-Que > Que (Fig. 3I). Finally, considering the dynamic pH variations under physiological conditions and to systematically evaluate the long-term ion release behavior and stability of Co-Que and CoMg-Que nanomaterials, ICP-OES was employed to monitor the release kinetics of Co^2+^ and Mg^2+^ from both materials (50 μg/ml) in SBF at pH 7.4 and 6.0 over 30 days. The results indicated that ions in both materials exhibited a sustained and slow-release behavior, with no abrupt burst-release peaks observed throughout the entire experimental period (Fig. 3G and H). Notably, under pH 6.0 conditions, the cumulative release amounts of Co^2+^ and Mg^2+^ were slightly higher and the release rates were moderately faster than those observed under pH 7.4 conditions. This phenomenon may be associated with slight dissociation of the coordination structure on the material surface in relatively acidic environments. Such a mild and sustained ion release profile is highly important for maintaining the long-term biological functionality of the material in vivo.

### Assessment of antibacterial efficacy and mechanism

**Fig. 4.**
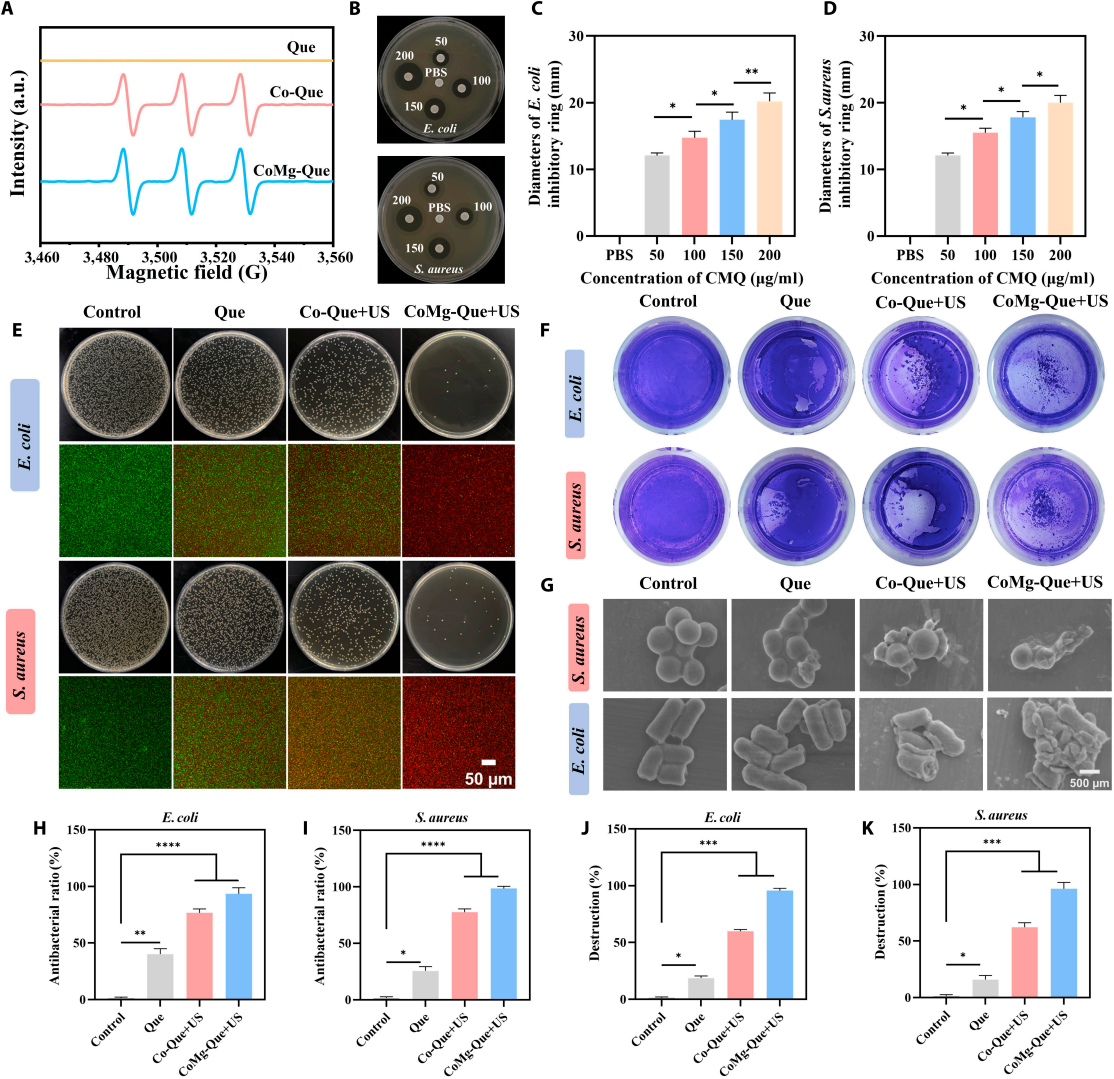
Antibacterial properties. (A) TEMP/^1^O_2_ ESR spectra of Que, Co-Que, and CoMg-Que (50 μg/ml). (B) Inhibition zone photographs of *E. coli* and *S. aureus* treated with different concentrations of CoMg-Que (50 to 200 μg/ml). (C and D) Quantitative evaluation of inhibition zone diameters for *E. coli* and *S. aureus* after treatment with various concentrations of CoMg-Que. (E, H, and I) Colony counting, live/dead staining, and antibacterial rate analysis of *E. coli and S. aureus* under different treatments. (F, J, and K) Biofilm formation assessed by crystal violet staining and corresponding disruption rates under different treatments. (G) SEM images of *E. coli* and *S. aureus* following different treatments. Data are presented as mean ± SD (*n* = 3). *P < 0.05, **P < 0.01, ***P < 0.001, and ****P < 0.0001.

To evaluate the ROS generation capability of the nanomaterials under ultrasonic stimulation, TEMP was used as a specific trapping agent for ^1^O_2_, and ESR analysis was performed to detect the ^1^O_2_ generation performance of free Que, Co-Que, and CoMg-Que under US irradiation (1.0 MHz, 1.5 W/cm^2^, 50% duty cycle). As presented in Fig. 4A, Co-Que and CoMg-Que exhibited the characteristic triplet ESR signal of ^1^O_2_ (1:1:1), and no obvious difference in signal intensity was observed, indicating that the 2 materials possessed similar ^1^O_2_ generation capabilities under ultrasonic activation. In contrast, free Que showed no detectable ^1^O_2_ signal under the same conditions, confirming its negligible contribution to US-triggered ROS production. These findings collectively suggest that the incorporation of Mg^2+^ did not significantly influence the ultrasonic ROS generation capability of the materials. Subsequently, a DPBF assay under ultrasonic irradiation (1.0 MHz, 1.5 W/cm^2^, 50% duty cycle) was conducted to quantitatively investigate the ^1^O_2_ generation rate. The dynamic consumption behavior of DPBF was monitored over 10 min (Fig. S7A). Consistent with the ESR results, the CoMg-Que group exhibited the most rapid DPBF decay rate, indicating the highest ^1^O_2_ generation efficiency under US activation. Pseudo-first-order kinetic fitting further demonstrated that CoMg-Que possessed the highest reaction rate constant (Fig. S7B), while its ROS generation rate remained comparable to that of Co-Que. In contrast, negligible DPBF consumption was detected in the free Que group, indicating that Que contributes minimally to sonodynamic ROS production. These results demonstrate that incorporation of Mg^2+^ and Que does not compromise sonodynamic activity while still enabling efficient US-triggered ^1^O_2_ generation. Based on the clarified ROS generation behavior and in order to determine experimental concentrations with both favorable biocompatibility and strong antibacterial efficacy, in vitro toxicity evaluations were first conducted to assess material safety. CCK-8 assay results (Figs. S8 to S10) demonstrated that cell viability gradually decreased with increasing nanoparticle concentration. Although different cell lines exhibited distinct sensitivities toward the materials, significant cytotoxicity was observed when the concentrations of Que, Co-Que, and CoMg-Que exceeded 100, 150, and 200 μg/ml, respectively. Moreover, the hemolysis assay (Fig. S11) demonstrated that CoMg-Que may damage erythrocyte membranes and induce hemolysis when the concentration exceeds 200 μg/ml. Meanwhile, the influence of US irradiation on cell viability was independently assessed in this work. As shown in Fig. S12, HUVECs and RAW264.7 cells were cocultured with the 200 μg/ml materials and subjected to US irradiation (1.0 MHz, 1.5 W/cm^2^, 50% duty cycle) for 10 min, followed by overnight incubation in a cell culture incubator. After live/dead staining, almost no dead cells (red fluorescence) were detected in the treatment groups. To further evaluate the potential biosafety of the cobalt-containing nanosystem, the cumulative release behavior of Co ions was monitored over 14 days at a material concentration of 200 μg/ml (Fig. S13). Compared with Co-Que, CoMg-Que exhibited relatively lower cumulative Co ion release during the 14-day period, thereby potentially improving the biosafety profile of the nanosystem. Importantly, the maximum cumulative Co ion release of CoMg-Que in SBF over 14 days was only 3.40 ppb, which remained far below concentrations generally associated with cobalt-related toxicity [33]. In addition, the local administration strategy adopted in this study may further minimize the risk of systemic cobalt exposure. Based on the toxicity evaluation results, inhibition zone assays against *E. coli* and *S. aureus* were subsequently performed using CoMg-Que within the safe concentration range. The results (Fig. 4B to D) demonstrated that the antibacterial activity increased in a concentration-dependent manner. Considering both biocompatibility and antibacterial performance, 200 μg/ml was selected as the standardized experimental concentration for subsequent investigations, as this concentration ensured good cytocompatibility while simultaneously exhibiting strong antibacterial activity.

In the in vitro antibacterial experiments, the antibacterial activities of different materials were first evaluated using the standard colony counting method (Fig. 4E, H, and I). Although CoMg-Que and Co-Que exhibited no obvious difference in ROS generation under US irradiation, the CoMg-Que+US group showed the strongest antibacterial effect, followed by the Co-Que+US group. This difference may be related to the lower crystallinity of CoMg-Que, which facilitates release of larger amounts of metal ions (Fig. 2D). Bacterial live/dead staining further supported these findings: almost all bacteria in the control group displayed green fluorescence, whereas after treatment with Que, Co-Que+US, and CoMg-Que+US, the green fluorescence intensity of SYTO-9 gradually decreased while the red fluorescence intensity of PI progressively increased in both *S. aureus* and *E. coli*. This trend was highly consistent with the colony counting results. Figure 4G shows SEM images of the 2 bacterial strains after different treatments. Bacteria in the control group exhibited smooth and intact surfaces with clear boundaries and regular morphology. After treatment with Que and Co-Que+US, the bacteria displayed visible structural damage, including membrane shrinkage and collapse. In the CoMg-Que+US group, bacterial morphology became severely deformed, ruptured, and even fragmented. These observations suggest not only that, under ultrasonic synergistic conditions, CoMg-Que induces multitarget oxidative damage to bacterial membranes, organelles, and nucleic acids through spatiotemporally controlled ROS bursts, but also that the released metal ions further disrupt membrane integrity by neutralizing the negative charge on bacterial surfaces. The rapid oxidative attack of ROS together with the sustained action of released metal ions synergistically contributes to complete bacterial destruction and death. Under pathological conditions, bacteria frequently exist in the form of biofilms, which possess more complex structures and stronger resistance. Therefore, the biofilm elimination capability of the materials was further investigated (Fig. 4F). As shown in Fig. 4J and K, the disruption rates of Co-Que+US and CoMg-Que+US against *E. coli* biofilms reached 59.83% and 95.60%, respectively, whereas against *S. aureus* biofilms, they reached 62.20% and 96.17%, respectively. These findings indicate that CoMg-Que, under US assistance, possesses extremely strong biofilm eradication capability.

Considering that MRSA is one of the predominant pathogens involved in orthopedic implant-associated infections and chronic bone defect infections [34], the biofilm formed by MRSA is regarded as a critical factor contributing to persistent infection and failure of conventional therapies. At present, vancomycin remains the primary first-line antibiotic for systemic clinical treatment of MRSA infections [35]. Therefore, to further evaluate the therapeutic potential of the CoMg-Que composite against refractory biofilm infections, MRSA and its corresponding biofilm were selected as experimental models, with vancomycin serving as the control drug, to systematically investigate its antibacterial and anti-biofilm performance. Based on the minimum inhibitory concentration (MIC) of vancomycin against MRSA being 1 μg/ml [36], the concentration in this study was uniformly set at 200 μg/ml to provide a preliminary concentration-matched comparison under identical short-term exposure conditions, while acknowledging that this does not reproduce the clinical administration regimen of vancomycin, which depends on maintaining sustained therapeutic concentrations over time [37]. Figure S14A and B demonstrate that, at the same treatment concentration, CoMg-Que+US still exhibited the strongest antibacterial activity, followed by Co-Que+US. By contrast, under this short exposure period, vancomycin did not fully exert its antibacterial efficacy, which is characteristic of time-dependent antibiotics. Similarly, the biomass of biofilms treated with Co-Que+US was reduced by about 74.11%, whereas CoMg-Que+US produced a much more pronounced elimination effect, achieving a biofilm reduction rate exceeding 99.05%. In comparison, vancomycin treatment at the same concentration under identical short-term conditions exerted only limited effects on preformed mature biofilms, with a clearance rate of merely 27.74% (Fig. S14C and D).

Combined with the experimental findings of this study, ROS generated under US irradiation demonstrated unique antibacterial advantages. Compared with antibacterial mechanisms that rely solely on ion release or intrinsic ROS generation from the materials themselves, where the intensity and duration are often difficult to accurately regulate, US irradiation enables spatiotemporal control over ROS generation. This characteristic was clearly reflected in the present study: by modulating US parameters, bursts of high-concentration ROS could be generated at specific locations and time points, thereby synergizing with ion release from CoMg-Que to achieve potent antibacterial effects. The severe deformation and rupture of bacterial cells, together with the nearly complete elimination of biofilms observed experimentally, provide direct evidence for this synergistic antibacterial mechanism. However, comparison between ROS therapy under short-term exposure and vancomycin treatment inherently has certain limitations. As a time-dependent antibiotic, the antibacterial efficacy of vancomycin depends on maintaining blood concentrations above the MIC for prolonged periods rather than achieving high peak concentrations [38]. Standard in vitro susceptibility assays and clinical therapeutic outcomes generally require continuous exposure [39], whereas the US treatment duration in this study was relatively brief. Consequently, such comparisons inherently favor burst-action antibacterial modalities such as ROS generation and do not represent a direct comparison of clinical efficacy. A more appropriate comparison should be conducted under pharmacologically optimized conditions for each therapeutic strategy, such as prolonged vancomycin incubation or pharmacokinetic simulation models. Mechanistically, the 2 antibacterial approaches differ substantially: vancomycin requires sustained high concentrations to inhibit bacterial cell wall synthesis over multiple bacterial generations [40], whereas CoMg-Que activated by US rapidly generates high concentrations of ROS through SDT [41], thereby inducing multitarget oxidative damage to cell membranes, proteins, and DNA to achieve rapid and nonspecific bactericidal activity. This multipathway oxidative damage acts rapidly and may partially circumvent conventional antibiotic resistance mechanisms. Considering these limitations, the superior antibacterial performance of CoMg-Que+US should be interpreted as demonstrating strong potential for rapid biofilm disruption rather than serving as a direct substitute for systemic vancomycin therapy. Future investigations should incorporate more clinically relevant control conditions, such as prolonged vancomycin exposure or localized antibiotic delivery systems, to more accurately define its potential therapeutic advantages in clinical settings.

### Anti-inflammatory and immunomodulatory properties

**Fig. 5.**
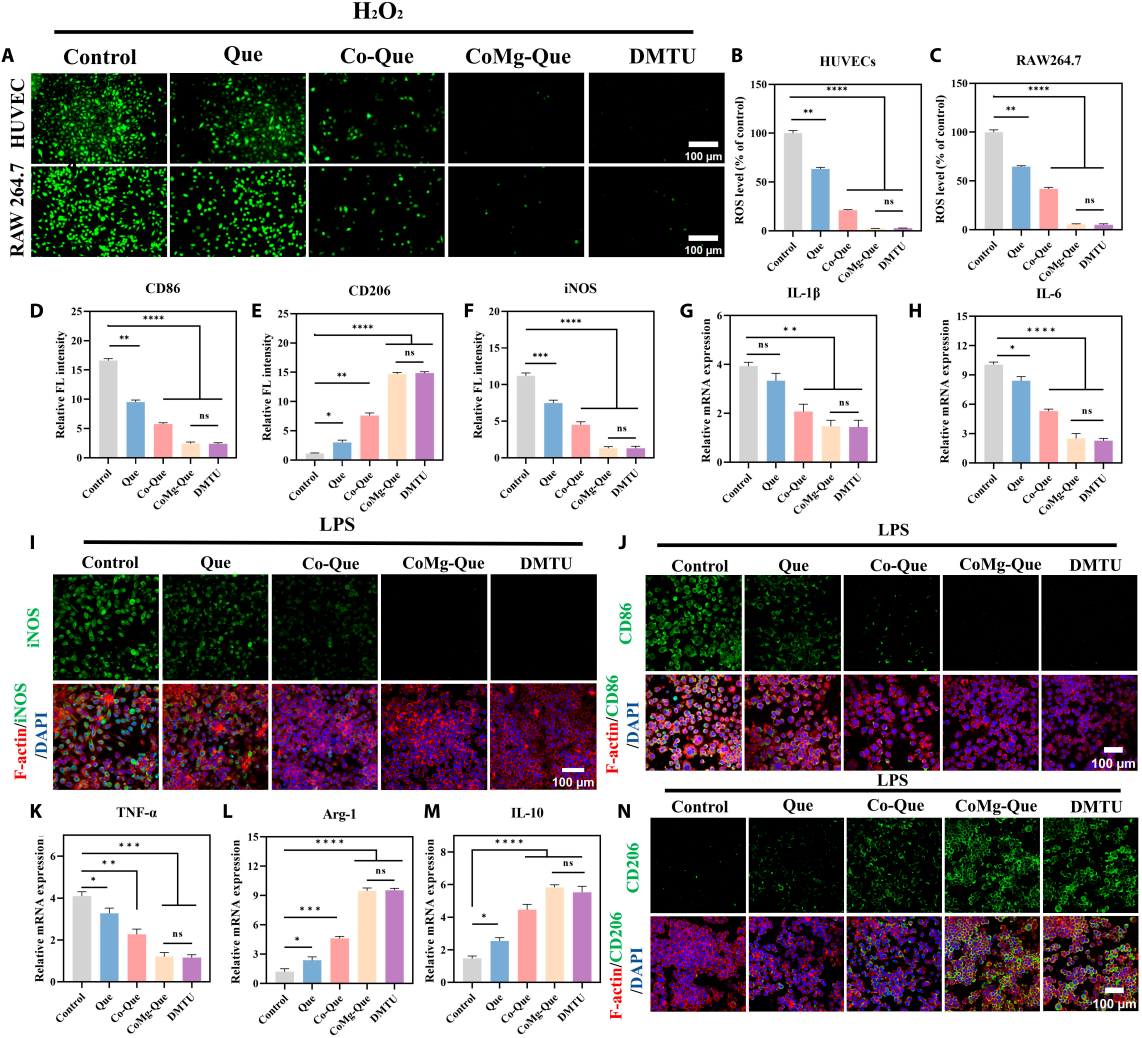
In vitro antioxidant and immunomodulatory effects. (A to C) Representative DCFH-DA staining images of HUVECs and RAW264.7 cells after H_2_O_2_ induction in different groups, together with corresponding statistical analyses. (D to F) Quantitative analysis of iNOS, CD86, and CD206 immunofluorescence staining. (G) IL-1β, (H) IL-6, (K) TNF-α, (L) Arg-1, and (M) IL-10 mRNA expression levels in LPS-stimulated RAW264.7 cells treated with different materials. (I, J, and N) Expression of iNOS, CD86, and CD206 in LPS-induced RAW264.7 cells under various treatments. All data are expressed as mean ± SD (*n* = 3). ns indicates *P* > 0.05, *P < 0.05, **P < 0.01, ***P < 0.001, and ****P < 0.0001.

Successful bone regeneration relies on the timely transition from the inflammatory stage to the bone formation phase. However, this process is frequently disrupted by persistent oxidative stress caused by excessive ROS accumulation. ROS not only activate osteoclast-associated pathways and accelerate bone resorption, but also drive immune cells toward a pro-inflammatory phenotype, thereby maintaining an inflammatory microenvironment unfavorable for tissue regeneration [42]. Therefore, efficient regulation of ROS levels together with restoration of immune balance is highly important for effective bone defect repair. To investigate the intracellular ROS-scavenging capability of different materials, the clinically approved ROS scavenger *N*,*N*′-dimethylthiourea (DMTU) [43] was employed as a positive control in this study. HUVECs and RAW264.7 cells were stimulated with H_2_O_2_ to establish an oxidative stress model, and after coculture with different materials, the regulatory effects of each nanomaterial and DMTU on intracellular ROS levels were compared using DCFH-DA staining. As shown in Fig. 5A to C, CoMg-Que exhibited a strong ability to reduce intracellular ROS levels compared with the other materials, with no statistically significant difference relative to DMTU. Based on these findings demonstrating the prominent intracellular ROS-scavenging capability of CoMg-Que, and considering the central role of ROS in immune cell polarization, the regulatory effect of this material on macrophage phenotypic transformation was further systematically evaluated to elucidate its potential role in inflammatory microenvironment remodeling and immune regulation. Immunofluorescence (IF) staining results (Fig. 5I, J, and N) revealed that, following LPS stimulation, macrophages predominantly underwent M1-type polarization, characterized by high expression levels of iNOS and CD86. However, after coincubation with different materials, macrophage phenotypes changed substantially: the expression levels of iNOS and CD86 progressively decreased, whereas the anti-inflammatory marker CD206 showed a corresponding increase. Among all groups, the CoMg-Que treatment group exhibited the most pronounced effect, significantly promoting macrophage polarization from the M1 phenotype toward the M2 phenotype (Fig. 5D to F), with an immunoregulatory effect comparable to that of the positive control group (DMTU). To further confirm the immunomodulatory effect suggested by IF staining, the expression levels of macrophage polarization-related genes were subsequently analyzed by qRT-PCR. The results demonstrated that the expression of M1-associated pro-inflammatory cytokines, including TNF-α (Fig. 5K), IL-1β (Fig. 5G), and IL-6 (Fig. 5H), was significantly down-regulated. In contrast, expression levels of M2-related markers, including interleukin-10 (IL-10, Fig. 5M) and arginase-1 (Arg-1, Fig. 5L), were markedly elevated. This trend was highly consistent with the IF staining observations, indicating that CoMg-Que can effectively direct macrophages from a pro-inflammatory M1 phenotype toward a reparative M2 phenotype.

Collectively, the anti-inflammatory and immunomodulatory properties of CoMg-Que may be attributed to its unique intrinsic catalytic characteristics. The controlled release of Co^2+^ and Mg^2+^ confers SOD- and CAT-like enzyme-mimetic activities upon the material, enabling cascade elimination of •O_2_^–^ and H_2_O_2_ and thereby effectively reducing oxidative stress at its source. Through regulation of intracellular redox homeostasis, CoMg-Que not only suppresses excessive inflammatory responses but also promotes macrophage polarization from the pro-inflammatory M1 phenotype toward the pro-regenerative M2 phenotype. Consequently, this material actively reconstructs a regeneration-supportive microenvironment rather than functioning merely as a passive anti-inflammatory agent, highlighting its considerable potential as a multifunctional platform integrating antibacterial and immunomodulatory functions for bone repair.

### Pro-angiogenic and osteogenic capabilities

Angiogenesis and osteogenic repair are not isolated biological events; rather, they represent a mutually dependent and synergistically regulated system during bone regeneration. These 2 biological processes are tightly interconnected through complex cellular and molecular signaling networks, collectively promoting bone tissue regeneration and remodeling [44,45].

**Fig. 6.**
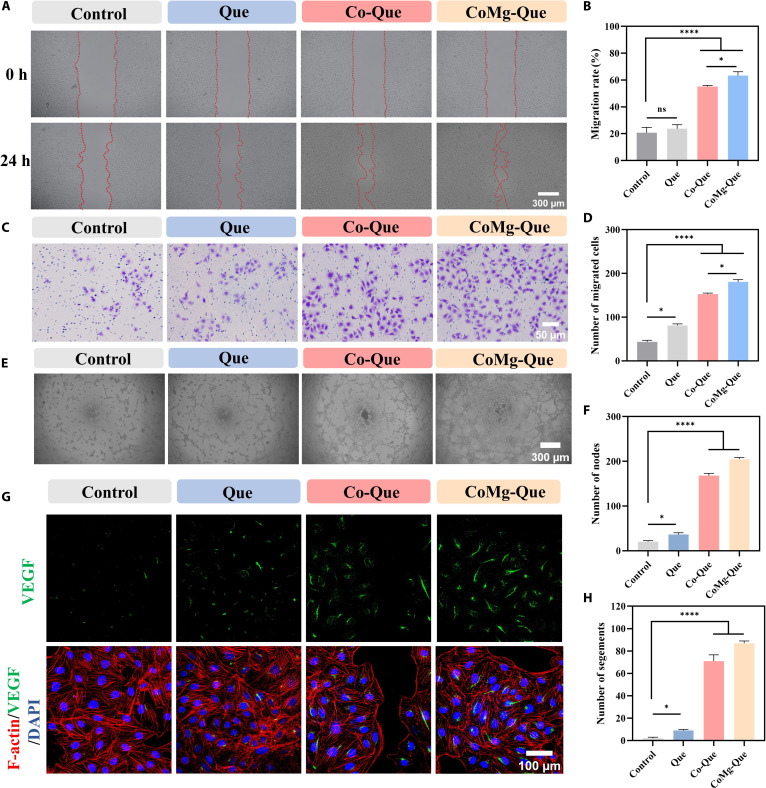
Angiogenic potential of HUVECs. (A and B) Scratch assay showing migration of HUVECs at 0 and 24 h in each group, together with quantitative analysis of migration rates. (C and D) Transwell migration assay of HUVECs after different treatments for 24 h and corresponding statistical analysis. (E) Tube formation assay of HUVECs after 6 h of treatment with different groups. (F and H) Quantitative analysis of node and segment numbers in the tube formation assay. (G) VEGF expression in HUVECs among different groups. All data are presented as mean ± SD (*n* = 3). ns represents *P* > 0.05, *P < 0.05, and ****P < 0.0001.

To systematically investigate the pro-angiogenic capability of the prepared materials, Transwell and scratch assays were initially conducted to evaluate their ability to promote endothelial cell migration. After 24 h of coculture with different materials, HUVECs in the CoMg-Que group exhibited the strongest scratch closure ability (Fig. 6A), followed by the Co-Que group (Fig. 6B). Similar trends were observed in the Transwell assay results (Fig. 6C and D), suggesting that Co^2+^ plays a critical role in mediating the pro-angiogenic activity of the material. Notably, incorporation of Mg^2+^ further enhanced this pro-angiogenic effect, implying that Mg^2+^ may synergistically amplify the biological activity induced by Co^2+^. This interpretation is consistent with previous reports demonstrating the direct stimulatory effects of Co^2+^ and Mg^2+^ on vascular endothelial cells, in which these metallic ions enhance endothelial proliferation and directional migration, thereby promoting neovascularization [46,47]. To further validate this hypothesis, tube formation assays (Fig. 6E to H), qRT-PCR (Fig. S15), and IF staining analyses (Fig. 6G) were subsequently performed. The results demonstrated that CoMg-Que exhibited excellent pro-angiogenic activity, with tube formation capability superior to that of the Co-Que group, further indicating that incorporation of Mg^2+^ can synergistically cooperate with Co^2+^ to enhance the angiogenic bioactivity of the material.

**Fig. 7.**
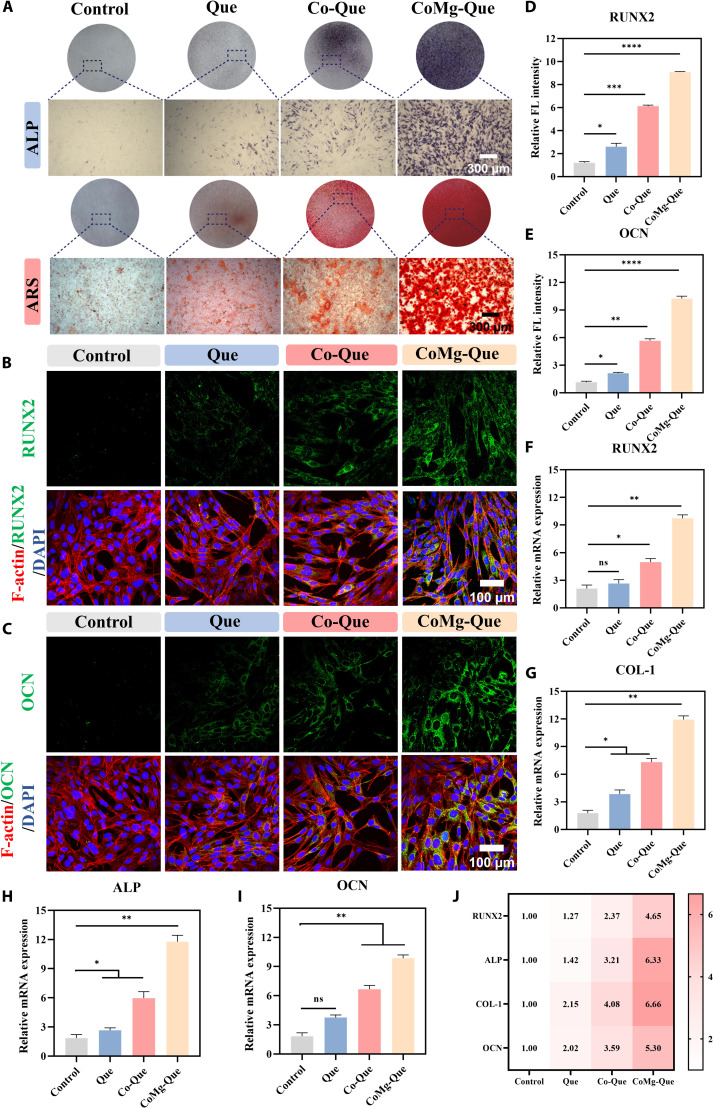
Osteoinductive properties. (A) Representative ALP staining images on day 14 and ARS staining images on day 21 in BMSCs for each group. (B and C) IF staining results of RUNX2 and OCN in BMSCs from different groups on day 14. (D and E) Quantitative analysis of RUNX2 and OCN IF staining results. (F) RUNX2, (G) COL-1, (H) ALP, and (I) OCN mRNA expression levels in BMSCs treated with different materials on day 14. (J) Heatmap visualization of BMSC mRNA expression profiles. All data are presented as mean ± SD (*n* = 3). ns represents *P* > 0.05, *P < 0.05, **P < 0.01, ***P < 0.001, and ****P < 0.0001.

Based on the excellent angiogenic performance of the material, its capability to promote osteogenesis in vitro was further investigated. BMSCs were cocultured with different materials, and osteogenic differentiation was evaluated using ALP and ARS staining (Fig. 7A). As shown in the figures, after 14 days of culture, the CoMg-Que group exhibited markedly stronger ALP staining intensity and a larger stained area compared with the control, Que, and Co-Que groups. By day 21, ARS staining demonstrated that the CoMg-Que group showed the most pronounced mineralized nodule formation in both staining intensity and distribution, indicating that this material can effectively enhance osteogenic differentiation and mineralization of BMSCs. Further molecular-level analyses were highly consistent with the phenotypic observations described above. qRT-PCR results revealed that, on day 14, the expression levels of representative osteogenesis-related genes, including COL-1, RUNX2, ALP, and OCN, were significantly up-regulated in the CoMg-Que group (Fig. 7F to J). Meanwhile, IF staining further demonstrated that the expression intensities of OCN and RUNX2 proteins in this group were also markedly higher than those in the Co-Que and control groups (Fig. 7B to E), further confirming the superior osteogenic differentiation-promoting capability of the CoMg-Que material toward BMSCs.

Taken together, the above findings demonstrate that the developed material possesses significant pro-angiogenic activity, in which Co^2+^ plays a major role in promoting endothelial cell migration and vascular network formation, while incorporation of Mg^2+^ further synergistically enhances angiogenesis and tube formation. Meanwhile, Mg^2+^ also improves the osteogenic performance of the system, significantly promoting osteogenic differentiation and mineralization of BMSCs. The synergistic regulation of angiogenesis and osteogenesis provided by CoMg-Que highlights its strong potential as a multifunctional biomaterial for vascularized bone regeneration.

### Exploring the pro-osteogenic mechanism

**Fig. 8.**
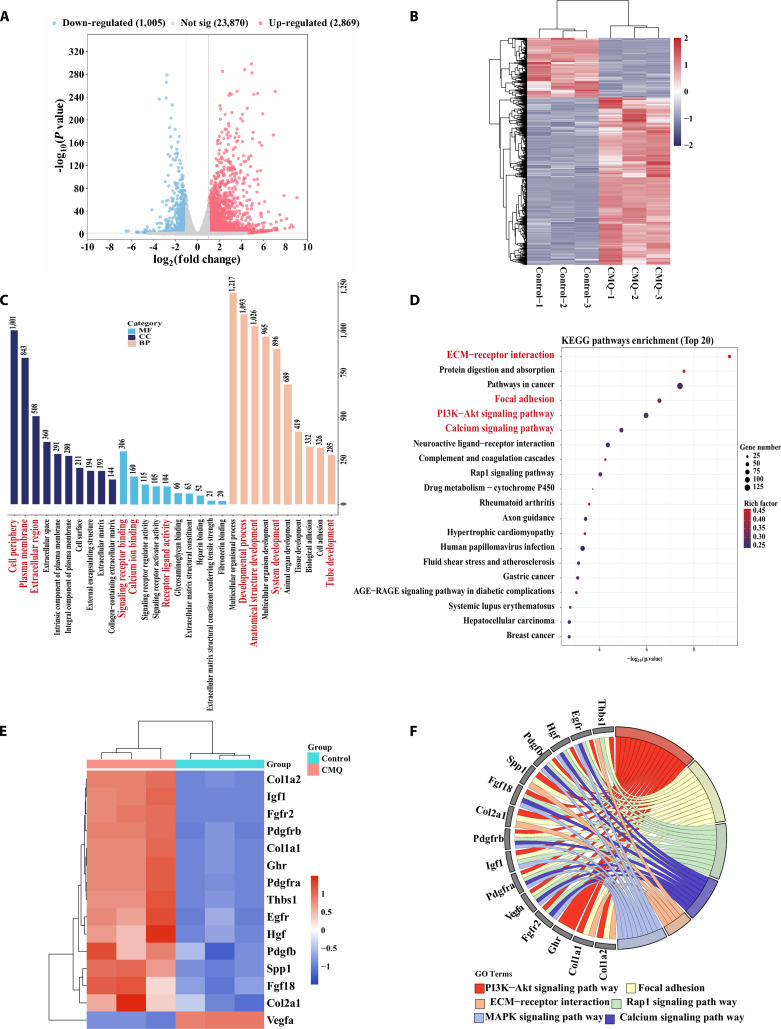
Transcriptomic sequencing analysis of BMSCs treated with CoMg-Que. (A) Volcano plot showing up-regulated and down-regulated genes. (B) Heatmap of DEGs. (C) Bar graph presenting GO enrichment analysis of up-regulated DEGs. (D) Bubble plot showing the top 20 KEGG enrichment pathways. (E) Heatmap displaying expression levels of major DEGs associated with the PI3K–Akt signaling pathway. (F) Circos plot of DEGs involved in multiple biological pathways.

Regeneration of IBD is a highly complex process that depends on the coordinated interplay of multiple cell types, intricate signaling pathways, and metal ion-mediated biological regulation. Following bone injury, repair and defense responses within the local microenvironment are rapidly activated, collectively orchestrating inflammatory regulation, tissue regeneration, and bone remodeling. To further elucidate the molecular mechanisms through which CoMg-Que promotes IBD repair, transcriptomic sequencing analysis was performed on cells from the control and CoMg-Que-treated groups. As shown in the volcano plot and DEG heatmap (Fig. 8A and B), compared with the control group, 2,869 genes were significantly up-regulated whereas 1,005 genes were markedly down-regulated in the CoMg-Que-treated group. GO enrichment analysis was subsequently conducted to investigate the biological functions associated with these DEGs. This analysis classifies genes into 3 major categories: cellular components (CC), biological processes (BP), and molecular functions (MF). Figure 8C presents the most significantly enriched GO terms within each category. In the CC category, the DEGs were mainly enriched in the cell periphery, plasma membrane, and extracellular regions. Their molecular functions were primarily associated with receptor binding, calcium ion binding, and ligand–receptor interactions. Within the BP category, these genes were predominantly related to developmental processes, anatomical structure development, and system development. Kyoto Encyclopedia of Genes and Genomes (KEGG) pathway enrichment analysis further identified the top 20 pathways with the highest enrichment scores (Fig. 8D). Notably, the DEGs were significantly enriched in pathways associated with cell adhesion and extracellular matrix interactions, including focal adhesion and extracellular matrix (ECM)–receptor interaction pathways, as well as osteogenesis-related signaling pathways such as calcium signaling and PI3K–Akt signaling. These pathways are critically involved in regulation of cell proliferation, adhesion, and bone matrix formation. To further clarify the potential regulatory network through which CoMg-Que promotes osteogenic differentiation, clustering analysis was performed on the major DEGs involved in the PI3K–Akt signaling pathway (Fig. 8E). According to their biological functions, these genes could generally be classified into 3 categories: genes associated with cell proliferation and growth factor signaling (including Igf1, Pdgfrb, and Fgfr2), genes involved in cell adhesion and extracellular matrix interactions (including Thbs1, Spp1, and Col1a1), and genes related to osteogenic differentiation and bone matrix formation (including Spp1, Col1a1, and Fgf18). In addition, the highly enriched DEGs identified through GO enrichment analysis together with their interaction networks are presented in Fig. 8F. To validate the transcriptomic findings, qRT-PCR was performed to quantify 4 representative DEGs (Col1a1, Spp1, Pdgfb, and Igf1). As shown in Fig. S16, all 4 genes were significantly up-regulated in the CoMg-Que-treated group compared to the control, consistent with the RNA-seq data.

Combining these transcriptomic findings with the previous in vitro experimental results, it can be inferred that, besides the direct biological contributions of Co^2+^, Mg^2+^, and Que, the osteogenic effect observed in this study is likely derived from the synergistic interaction between sustained ion release and a ROS-scavenging microenvironment. During bone regeneration, excessive ROS can inhibit osteoblast differentiation and induce apoptosis. CoMg-Que not only continuously releases osteogenic ions but also alleviates local oxidative stress through the antioxidant activity of Que, thereby establishing a favorable redox environment for bone formation. This dual mechanism—integrating ion-mediated signal activation (such as calcium signaling and PI3K–Akt pathway activation) together with ROS scavenging—may synergistically promote BMSC adhesion, proliferation, and osteogenic differentiation. Transcriptomic evidence supporting this interpretation includes the simultaneous enrichment of extracellular matrix- and cell adhesion-related genes, which benefit from reduced oxidative stress, together with genes directly involved in osteogenesis. Therefore, it can be speculated that CoMg-Que may enhance BMSC adhesion and proliferation through the synergistic effects of Co^2+^, Mg^2+^, and Que, while simultaneously establishing a ROS-scavenging microenvironment, thereby further activating the PI3K–Akt signaling pathway and ultimately promoting osteogenic differentiation and new bone formation.

### In vivo antibacterial and osteogenic performance

**Fig. 9.**
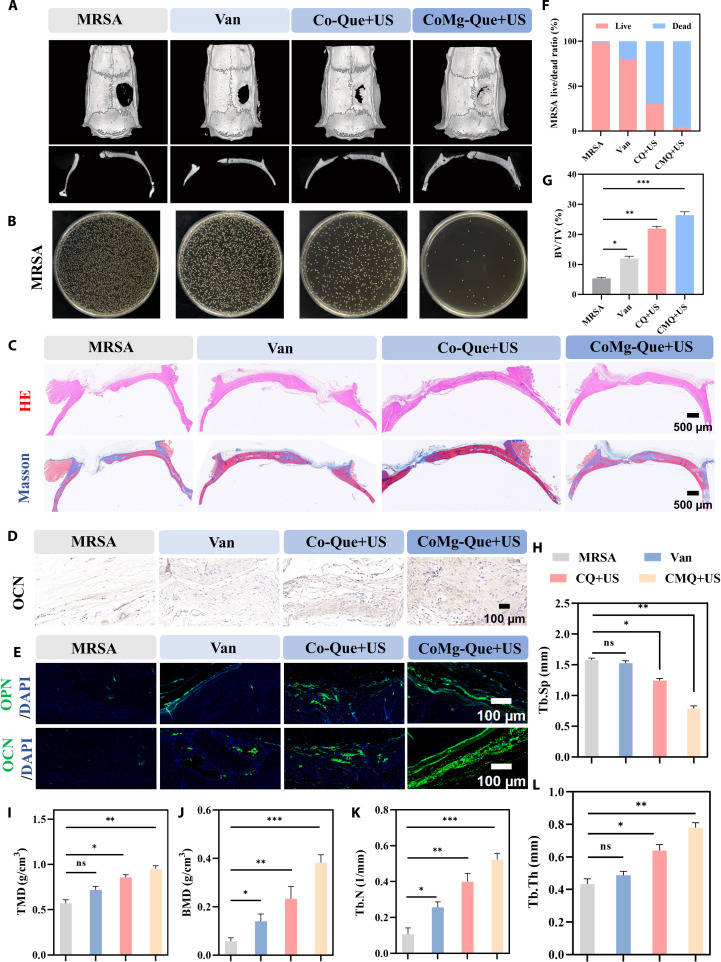
In vivo antibacterial and bone regeneration capabilities. (A) Micro-CT analysis of calvarial bone specimens from different groups at 8 weeks after treatment. (B) Plate counting images of MRSA collected from wound sites on day 3 in each group. (C) Representative H&E and Masson’s trichrome staining images of calvarial specimens harvested at 8 weeks. (D) IHC staining results of OCN in calvarial specimens at 8 weeks. (E) IF staining images of OPN and OCN in calvarial sections at 8 weeks. (F) Quantitative analysis of MRSA colonies on day 3. (G) Quantitative micro-CT parameters including BV/TV, (H) Tb.Sp, (I) TMD, (J) BMD, (K) Tb.N, and (L) Tb.Th. All data are presented as mean ± SD (*n* = 3). ns represents *P* > 0.05, *P < 0.05, **P < 0.01, and ***P < 0.001.

The sonodynamic therapeutic efficacy of CoMg-Que for treatment of IBD was further evaluated using an MRSA-infected critical-sized cranial defect model in rats. A 5-mm-diameter defect was generated in the right parietal bone of SD rats using a trephine drill, and MRSA suspension was immediately injected into the defect region to establish localized infection. Three days later, the original incision was reopened, and PBS (MRSA group), Co-Que (Co-Que+US group), or CoMg-Que (CoMg-Que+US group) was administered into the defect site. Subsequently, the Co-Que+US and CoMg-Que+US groups were exposed to US irradiation (1.5 W/cm^2^, 50% duty cycle, 1 MHz) for 10 min. Meanwhile, a Van group was included as the clinical gold-standard control. On day 3 post-infection, vancomycin (40 mg/kg) was intravenously administered via tail vein injection according to dosing regimens reported in previous studies [48,49]. Exudates collected from the defect area were subsequently analyzed to assess in vivo antibacterial efficacy. As shown in Fig. 9B, a large number of bacteria were observed in the defect regions of both the MRSA and Van groups. In contrast, bacterial counts were markedly reduced in the Co-Que+US group, whereas only a few residual bacteria were detected in the CoMg-Que+US group. Quantitative analysis further demonstrated antibacterial rates of 69.35% and 95.24% in the Co-Que+US and CoMg-Que+US groups, respectively (Fig. 9F). This tendency was highly consistent with the in vitro antibacterial results, further confirming the potent in vivo antibacterial activity of CoMg-Que under US activation. Micro-CT analysis performed 8 weeks after surgery (Fig. 9A) demonstrated that MRSA infection resulted in almost no new bone formation within the defect region. In contrast, different degrees of bone repair were observed in the Van, Co-Que+US, and CoMg-Que+US groups, among which the CoMg-Que+US group exhibited the most substantial defect closure and the smallest residual defect area. To further quantitatively compare bone regeneration among different groups, bone microstructural parameters including bone volume fraction (BV/TV), bone mineral density (BMD), tissue mineral density (TMD), trabecular number (Tb.N), trabecular separation (Tb.Sp), and trabecular thickness (Tb.Th) were systematically analyzed (Fig. 9G to L). The results revealed that the CoMg-Que group displayed the most favorable values across all evaluated parameters, confirming its superior efficacy in promoting repair of IBD. Histological examinations further supported these findings. H&E and Masson staining demonstrated extensive new bone formation within the defect area in the CoMg-Que+US group, whereas only minimal regeneration was observed in the MRSA group (Fig. 9C). Immunohistochemistry (IHC) staining further showed that expression of the osteogenic marker OCN was strongest in the CoMg-Que+US group (Fig. 9D). Consistently, IF analysis demonstrated significantly increased expression levels of the osteogenesis-related proteins OPN and OCN in this group compared with the other treatment groups (Fig. 9E), further confirming its strong osteogenic capacity even under infectious conditions. Finally, histological evaluation of major organs including the heart, liver, spleen, lungs, and kidneys after 8 weeks revealed no obvious tissue injury or inflammatory lesions, indicating favorable systemic biosafety of the developed materials (Fig. S17).

It should be noted that, although the in vivo study was conducted with a limited sample size (*n* = 3 per group), consistent trends were observed across all animals within each group. In accordance with the 3R principles (Replacement, Reduction, and Refinement) and ethical requirements, the study was designed to minimize animal use while maintaining experimental validity. Therefore, these in vivo findings should be interpreted with appropriate caution, and further studies with larger cohorts are required to validate the long-term efficacy and statistical robustness.

Overall, in an MRSA-infected rat cranial defect model, CoMg-Que combined with US treatment exhibited pronounced synergistic antibacterial and bone-regenerative effects. The system not only efficiently eradicated bacteria but also significantly promoted new bone formation and improved bone microarchitecture, while maintaining favorable biosafety, indicating its considerable potential for the treatment of IBD.

## Conclusion

Infectious conditions markedly impair the physiological repair of bone defects, making the originally coordinated and multistage bone regeneration process more complicated and inefficient. To address this clinical challenge, a sonosensitive nanozyme, CoMg-Que, was successfully developed in the present study. Under US stimulation, this nanoplatform efficiently generated ROS to achieve sonodynamic antibacterial therapy, thereby rapidly eliminating pathogens while reducing the potential risk of antibiotic resistance. Following cessation of US irradiation, its SOD- and CAT-like enzymatic activities further scavenged excessive ROS, thereby restoring local redox balance and regulating the inflammatory microenvironment. Meanwhile, the synergistic interactions among Co^2+^, Mg^2+^, and Que promoted angiogenesis and established a favorable biological microenvironment for bone regeneration. Notably, CoMg-Que significantly enhanced the osteogenic differentiation capability of BMSCs, which may be closely associated with activation of the PI3K–Akt signaling pathway. In vivo investigations further demonstrated that, in an MRSA-infected rat calvarial defect model, this nanosystem exhibited strong antibacterial activity, effective inflammatory regulation, and enhanced bone regeneration, while also showing good biocompatibility without detectable toxic effects or pathological abnormalities in normal tissues. Although the relatively slow Co ion release profile together with the short-term biosafety evaluation suggested acceptable biocompatibility, the long-term biodegradation behavior, systemic distribution, and possible cobalt accumulation of this cobalt-containing nanosystem still require more comprehensive investigation. Therefore, future studies focusing on chronic biosafety, metabolic clearance, and long-term in vivo evaluation will be essential for facilitating its clinical translation. Overall, this work presents a multifunctional nanoplatform capable of integrating sonodynamic antibacterial activity, oxidative stress regulation, and bone regeneration promotion, thereby providing a new strategy and theoretical foundation for precise IBD treatment and the development of next-generation bone repair biomaterial.

## Data Availability

The datasets used and/or analyzed during the current study are available from the corresponding authors upon reasonable request.
