# Supplementary material for: Sonodynamic CoMg-Quercetin Nanozyme for Antibacterial Therapy and Multifunctional Bone Regeneration in Infectious Bone Defects
Source: Biomater Res. 2026 Jul 14;30:0396. doi: 10.34133/bmr.0396 (PMC13365584; doi:10.34133/bmr.0396)
Supplement: Supplementary 1 — Figs. S1 to S17 Tables S1 and S2 [file bmr.0396.f1.docx]

***Supplementary Material***

**Sonodynamic CoMg-Quercetin Nanozyme for Antibacterial Therapy and Multifunctional Bone Regeneration in Infectious Bone Defects**

Yurong Xu^1,2,†^, Jingyu Yan^1,2,†^, Lihong Zhou^1,2,†^, Chenying Cui^1,2^, Kaifang Zhang^1,2^, Kun Liu^1,2^, Xiuping Wu^1,2,*^, Bing Li^1,2,*^

^1^Shanxi Medical University School and Hospital of Stomatology, Taiyuan, 030001, Shanxi, China. ^2^Shanxi Province Key Laboratory of Oral Diseases Prevention and New Materials, Taiyuan, 030001, Shanxi, China.

*Address correspondence to: libing1975vip@163.com (B. L.); 77wxp@163.com (X.W.)

†. These authors have contributed equally to this work.

**Table**

**Table S1. Primer sequences for qRT-PCR.**

| **Gene** | **Forward primer** | **Reverse primer** |
| --- | --- | --- |
| Arg-1 | CCCAGCTTGTCTACTTCAGTCATG | GGCAACCTGTGTCCTTTCTCCT |
| IL-6 | ATA ACC ACC CCT GAC CCA AC | CCC ATG CTA CAT TTG CCG AA |
| IL-10 | GGAAGACAATAACTGCACCCACT | CAACCCAAGTAACCCTTAAAGTCC |
| IL-1β | TGTGTTTTCCTCCTTGCCTCTGAT | TGCTGCCTAATGTCCCCTTGAAT |
| TNF-α  CD31  VEGF  RUNX2  COL-1  ALP  OCN  Col1a1  Spp1  Pdgfb  Igf1  GAPDH | CTTGTTGCCTCCTCTTTTGCTTA  CCAAAGCCAGTAGCATCATGGTC  AACTTTCTGCTGTCTTGG  GACTGTGGTTACCGTCATGGC  TAG TCT GTC CTG CGT CCT CTG  GACCTCCTCGGAAGACACTC  GTGCAGAGTCCAGCAAAGGT  GCTCCTCTTAGGGGCCACT  CAGTGATTTGCTTTTGCCTCC  CCAGAAAGAAGAGAGAGGACAA  CCTGTGGATGAGTGTTGCTT  GACTCATGACCACAGTCCATGC | CTTTATTTCTCTCAATGACCCGTAG  GGATGGTGAAAGTTGGCACAGG  ACTTCGTGATGATTCTGC  ACTTGGTTTTTCATAACAGCGGA  GAG TCT TTT GCT TCC TCC CAC  TGAAGGGCTTCTTGTCTGTG  TCCCAGCCATTGATACAGGT  CCACGTCTCACCATTGGGG  TCTGATCTTCTTACTGCTTCTG  GGAGTCCAGGTCAAAGAGAAAG  CGTAGTTCTTGTTTCCTGCACT  AGAGGCAGGGATGATGTTCTG |

**Table S2.** **Physicochemical characteristics of CoMg-Que.**

| **Sample**  **CoMg-Que** | **^1^Core size (nm)**  **155.89(****±5.71)** | **^2^Hydrodynamic size (nm)**  **182.43(±3.23)** | **^3^Zeta potential (mV)**  **-33.02(±2.12)** |
| --- | --- | --- | --- |

**Data are presented as mean ± SE (n = 3).**

**^1^Obtained from SEM images using the ImageJ software (n = 100).**

**^2, 3^Obtained from DLS.**

**
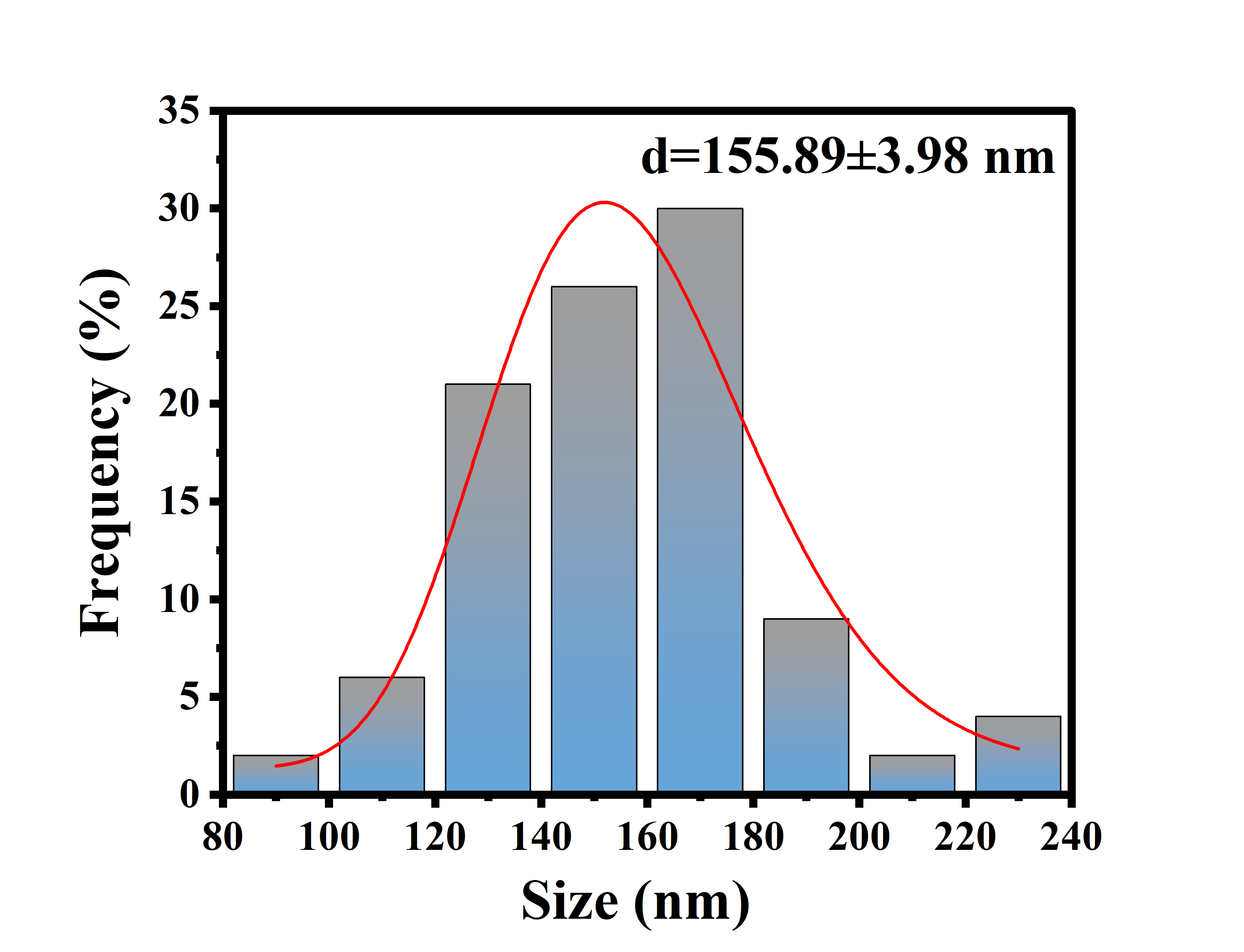
**

**Fig. S1 Core size distribution of CoMg-Que derived from the SEM image** **shown in Figure 1. (n = 100)**

**
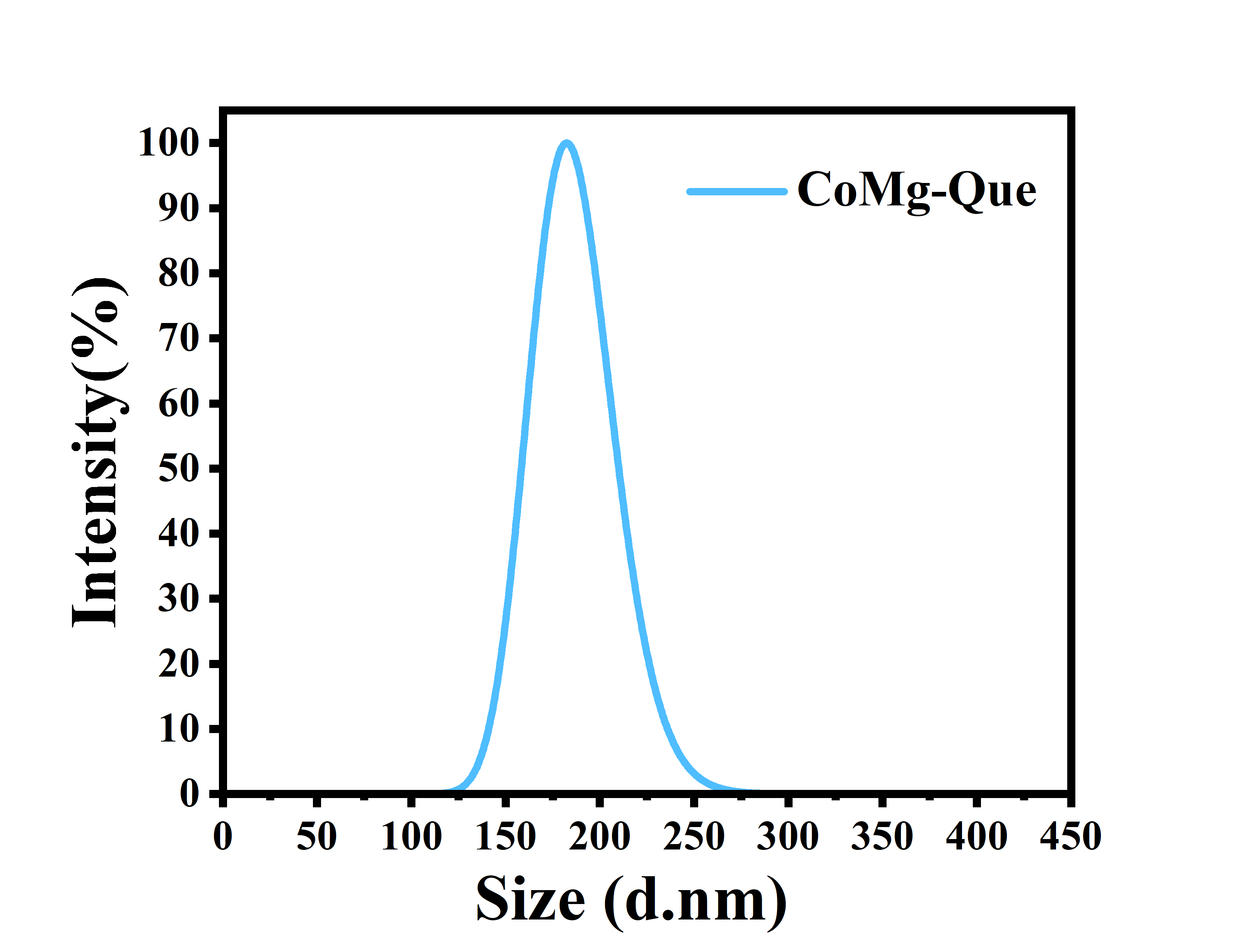
**

**Fig. S2 DLS size distribution profile of CoMg-Que dispersed in PBS.**

**
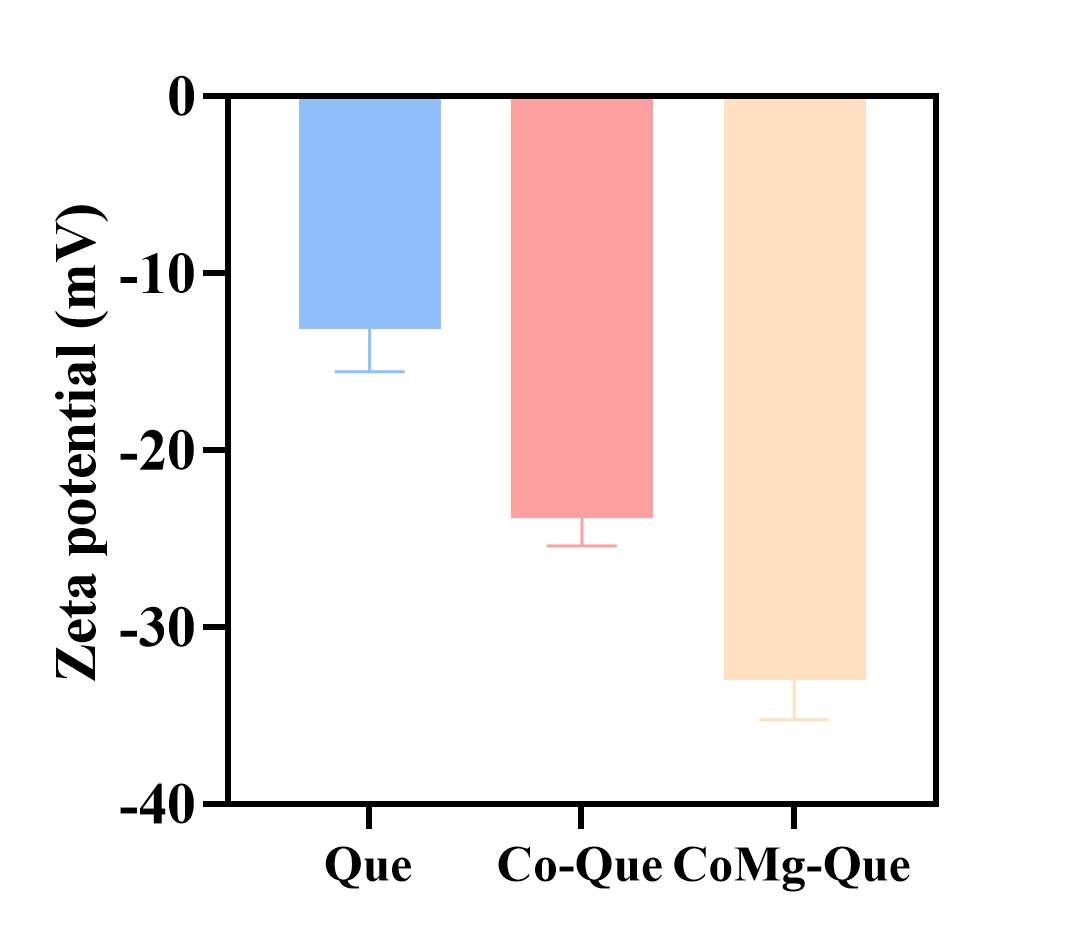
**

**Fig. S3 Zeta potential of Que, Co-Que, and CoMg-Que dispersed in PBS. Data are shown as mean ± SD (n = 3).**

**
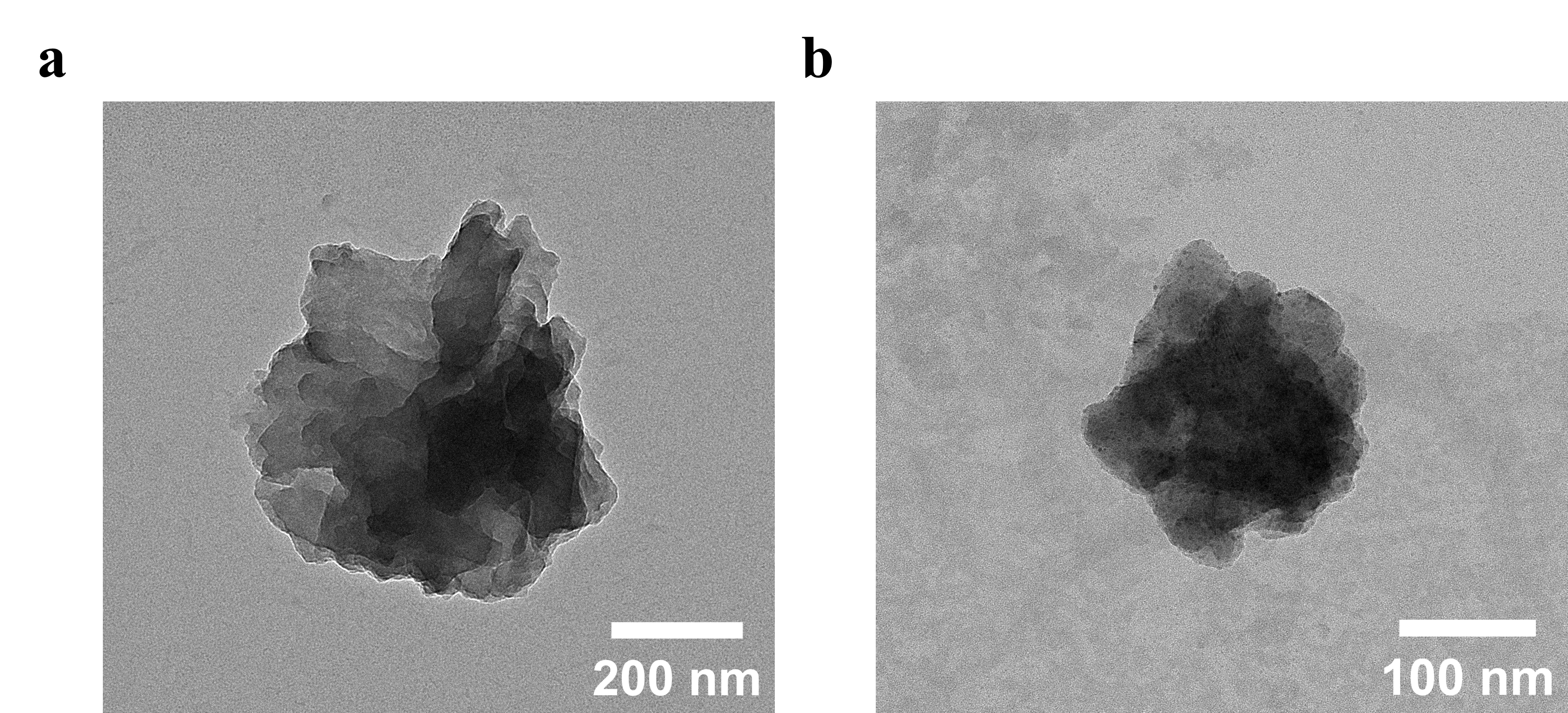
**

**Fig. S4 TEM images of Co-Que and CoMg-Que. (a) Co-Que (b) CoMg-Que (n = 3)**

**
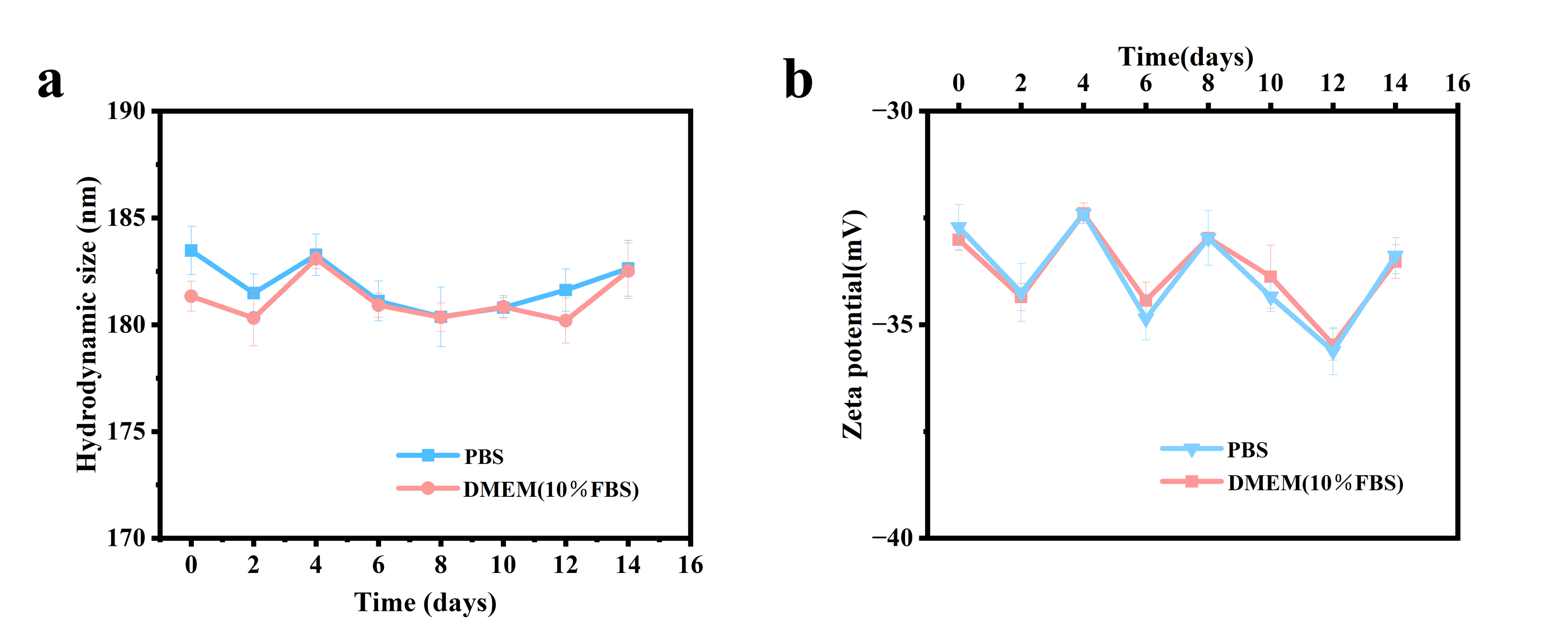
**

**Fig. S5 Colloidal stability of CoMg-Que nanozymes in physiological media at 37 °C. (a) Hydrodynamic diameter and (b) zeta potential of CoMg-Que dispersed in PBS and DMEM containing 10% FBS, monitored over 14 days. Data are presented as mean ± standard deviation (n = 3).**

**
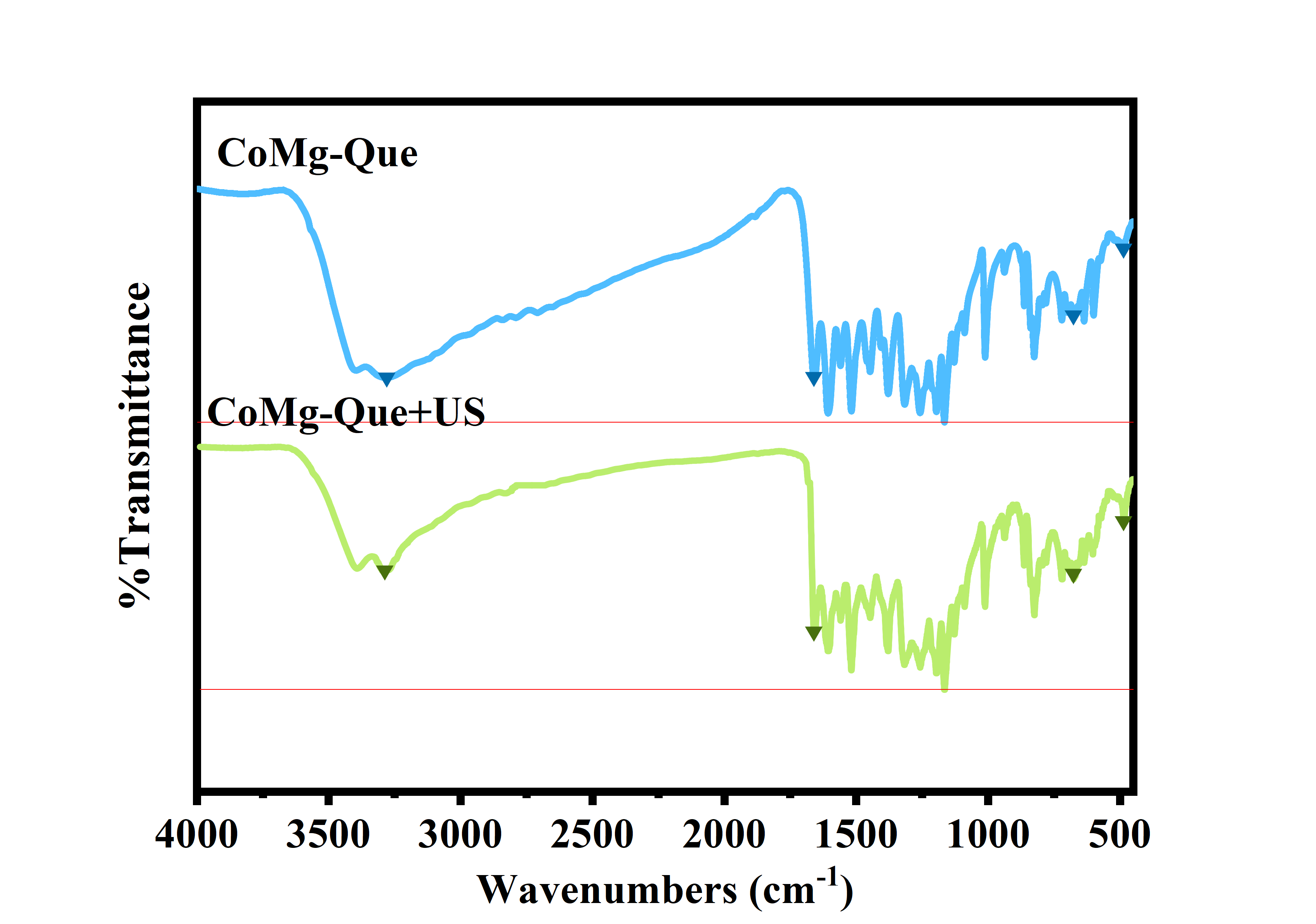
**

**Fig. S6** **FTIR spectra of CoMg-Que before and after US irradiation (10 min). (n = 3)**

**
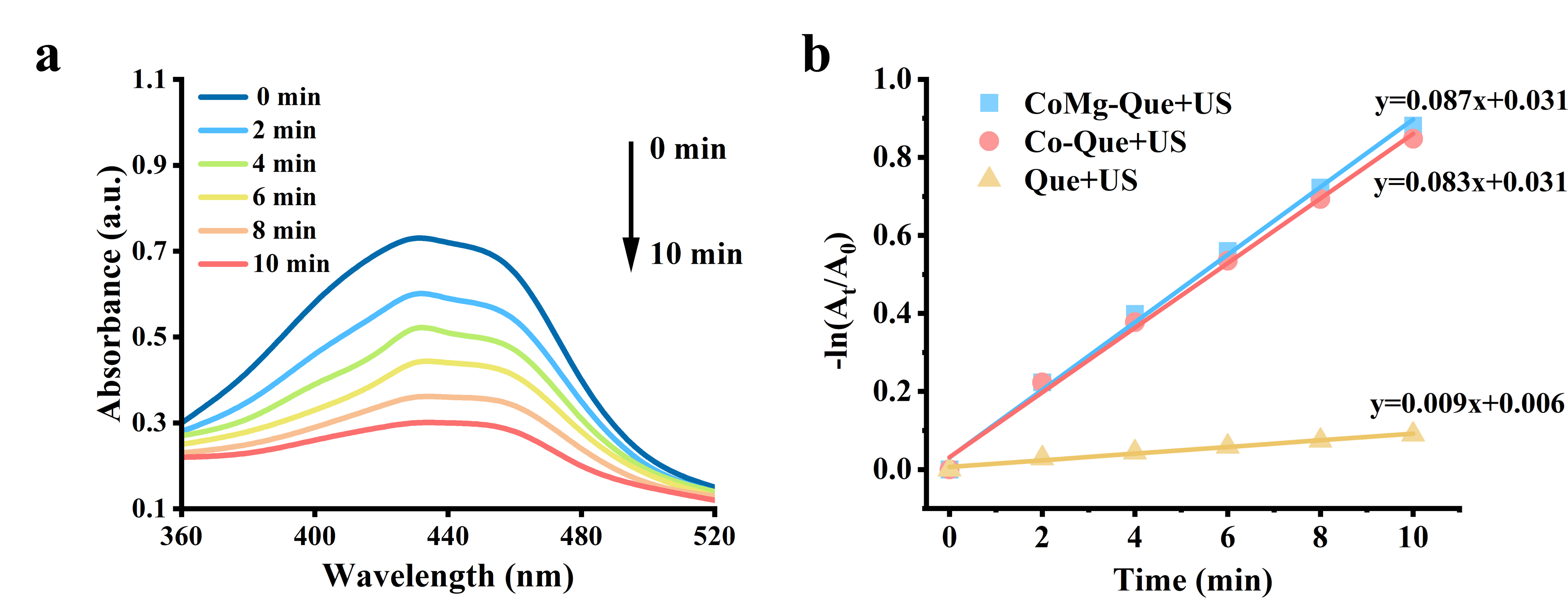
**

**Fig. S7a: Time-dependent absorbance changes of DPBF induced by CoMg-Que (50 μg/mL) under 10 min US irradiation; b:** **First-order rate constants of different materials (50 μg/mL) under 10 min US irradiation. (n = 3)**

**
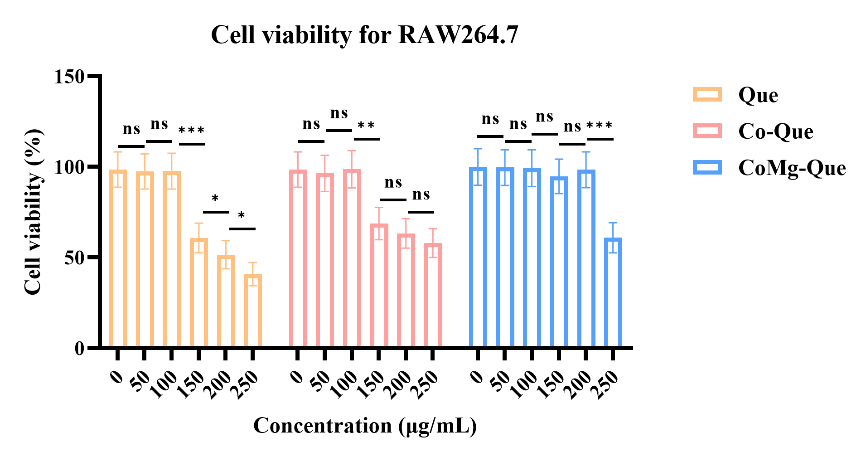
**

**Fig. S8 Study on the cytotoxicity of different concentrations of materials on RAW264.7 after 5 days of treatment.** **All data are presented as mean ± SD (n = 3). ns represents p > 0.05, * represents p < 0.05, ** represents p < 0.01, *** represents p < 0.001, and **** represents p < 0.0001.**

**
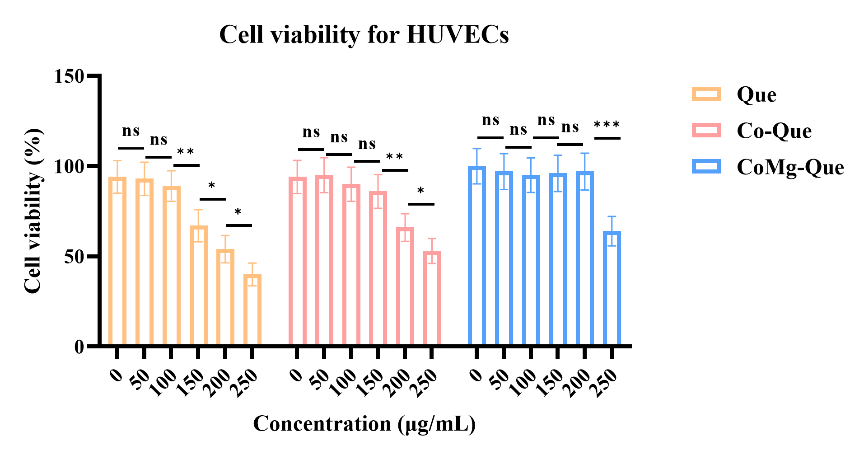
**

**Fig. S9 Study on the cytotoxicity of different concentrations of materials on HUVECs after 5 days of treatment.** **All data are presented as mean ± SD (n = 3). ns represents p > 0.05, * represents p < 0.05, ** represents p < 0.01, *** represents p < 0.001, and **** represents p < 0.0001.**

**
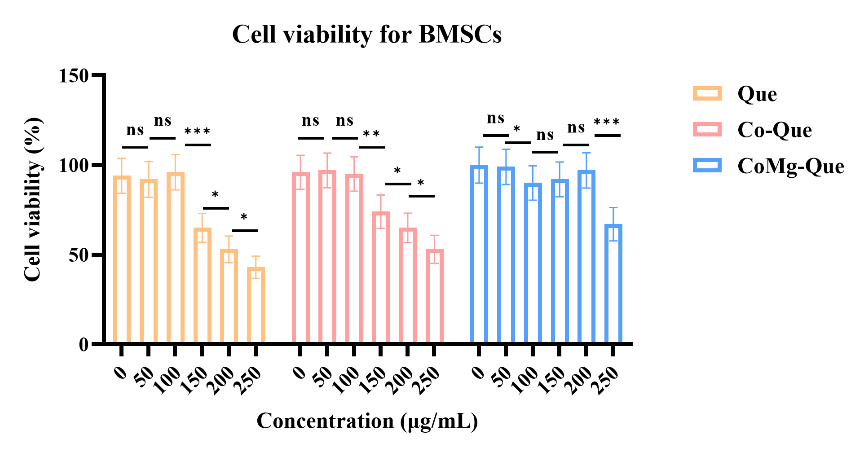
**

**Fig. S10 Study on the cytotoxicity of different concentrations of materials on BMSCs after 5 days of treatment.** **All data are presented as mean ± SD (n = 3). ns represents p > 0.05, * represents p < 0.05, ** represents p < 0.01, *** represents p < 0.001, and **** represents p < 0.0001.**

**
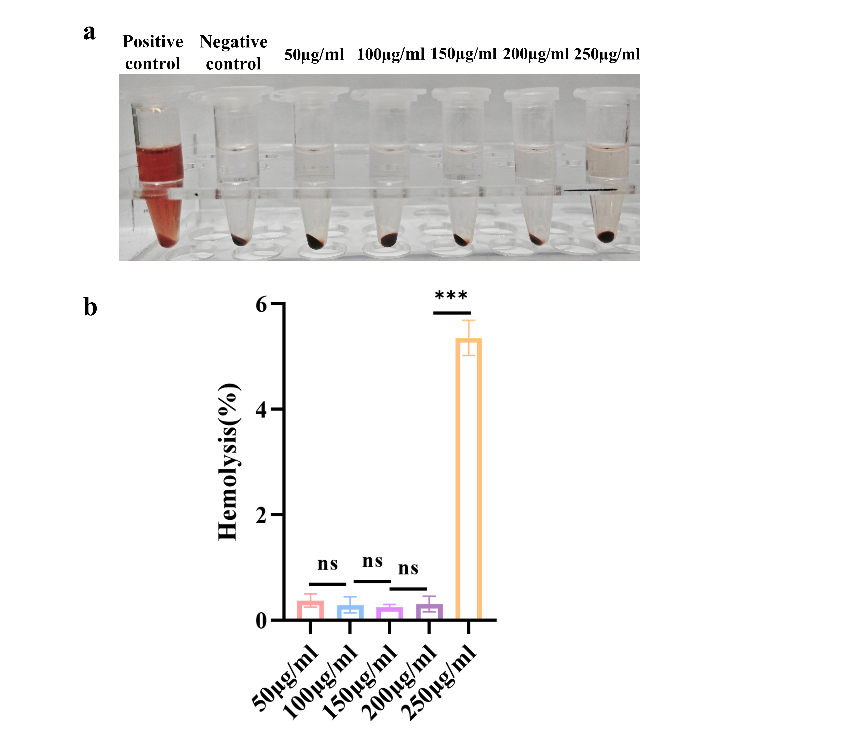
**

**Fig. S11 (a, b) Hemolysis test and hemolysis rate of CoMg-Que at various concentrations.** **All data are presented as mean ± SD (n = 3). ns represents p > 0.05, * represents p < 0.05, ** represents p < 0.01, *** represents p < 0.001, and **** represents p < 0.0001.**

**
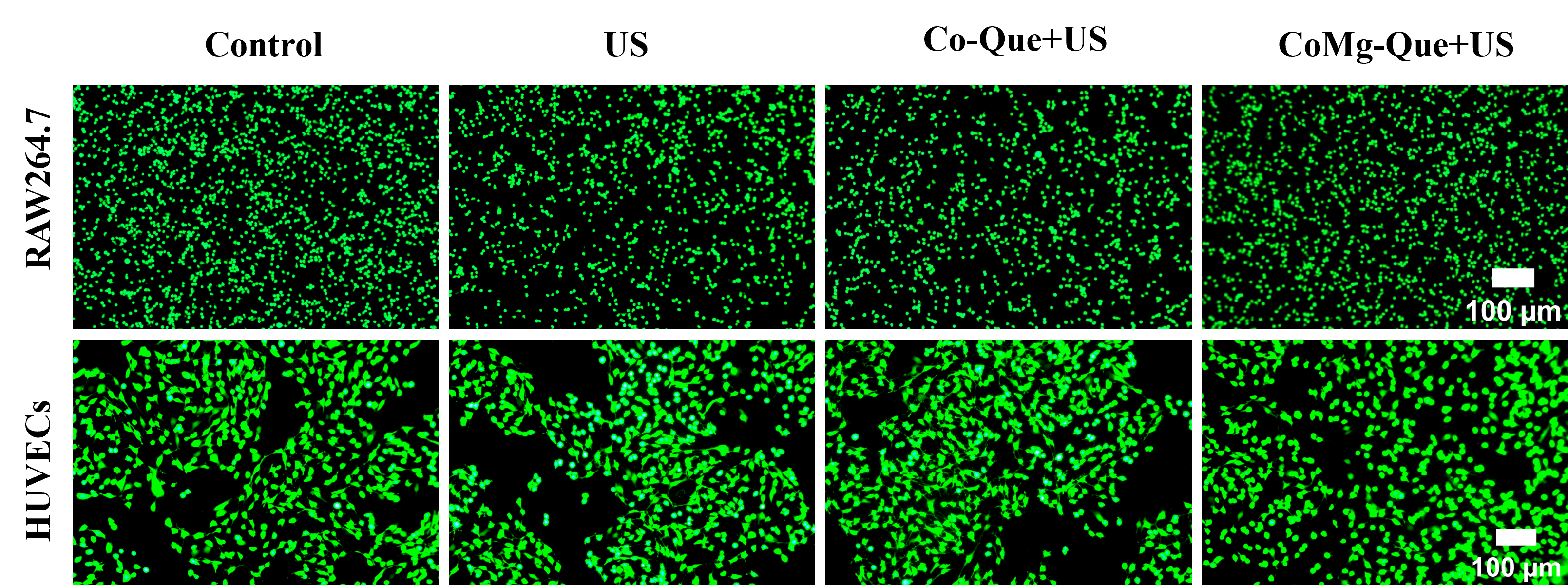
**

**Fig. S12 Live/dead staining images of RAW264.7 and HUVECs after 10 min US** **irradiation. (n = 3)**

**
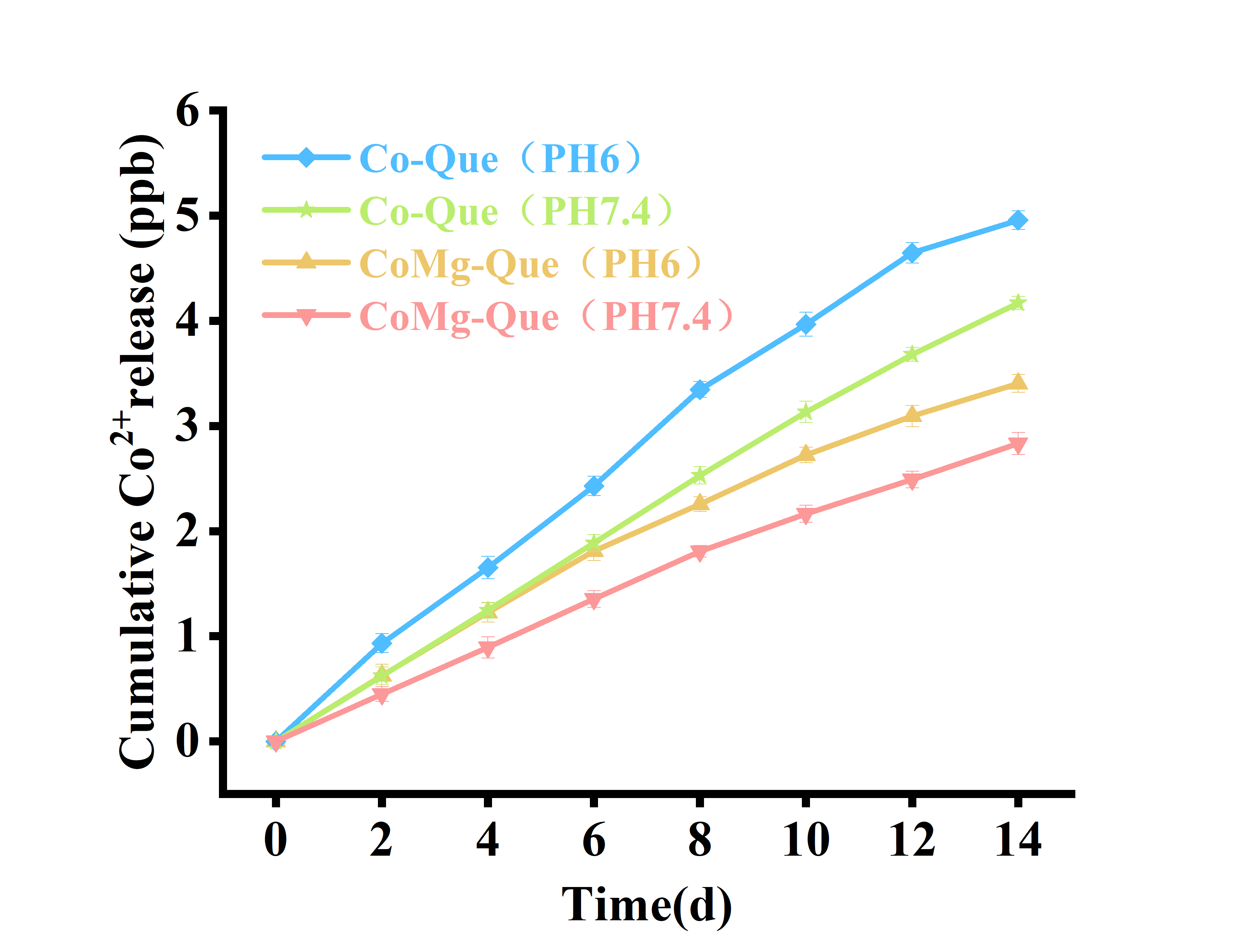
**

**Fig. S13 The cumulative release of Co^2+^ from Co-Que and CoMg-Que** **(200 μg/mL) in SBF (pH 6.0 and 7.4) over 14 days.** **All data are presented as mean ± SD (n = 3). ns represents p > 0.05, * represents p < 0.05, ** represents p < 0.01, *** represents p < 0.001, and **** represents p < 0.0001.**

**
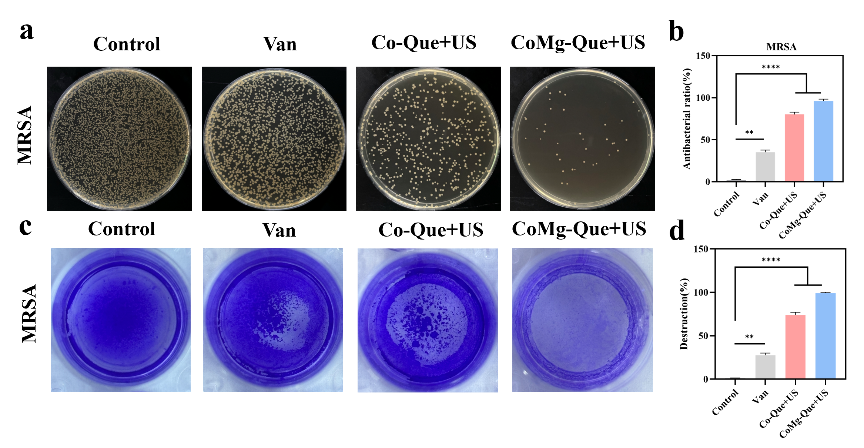
**

**Fig. S14 (a, b) Evaluation of the antibacterial performance of different materials against MRSA using the plate colony counting method, and calculation of their antibacterial rates. (c, d) Effects of different treatments on bacterial biofilms and their disruption rates.** **All data are presented as mean ± SD (n = 3). ns represents p > 0.05, * represents p < 0.05, ** represents p < 0.01, *** represents p < 0.001, and **** represents p < 0.0001.**

**
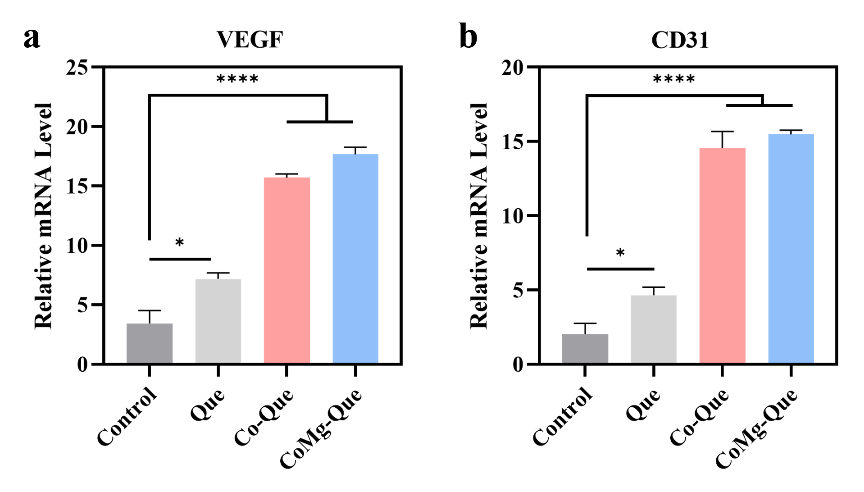
**

**Fig. S15 (a, b) VEGF and CD31** **mRNA expression levels in HUVECs treated with different materials.** **All data are presented as mean ± SD (n = 3). ns represents p > 0.05, * represents p < 0.05, ** represents p < 0.01, *** represents p < 0.001, and **** represents p < 0.0001.**

**
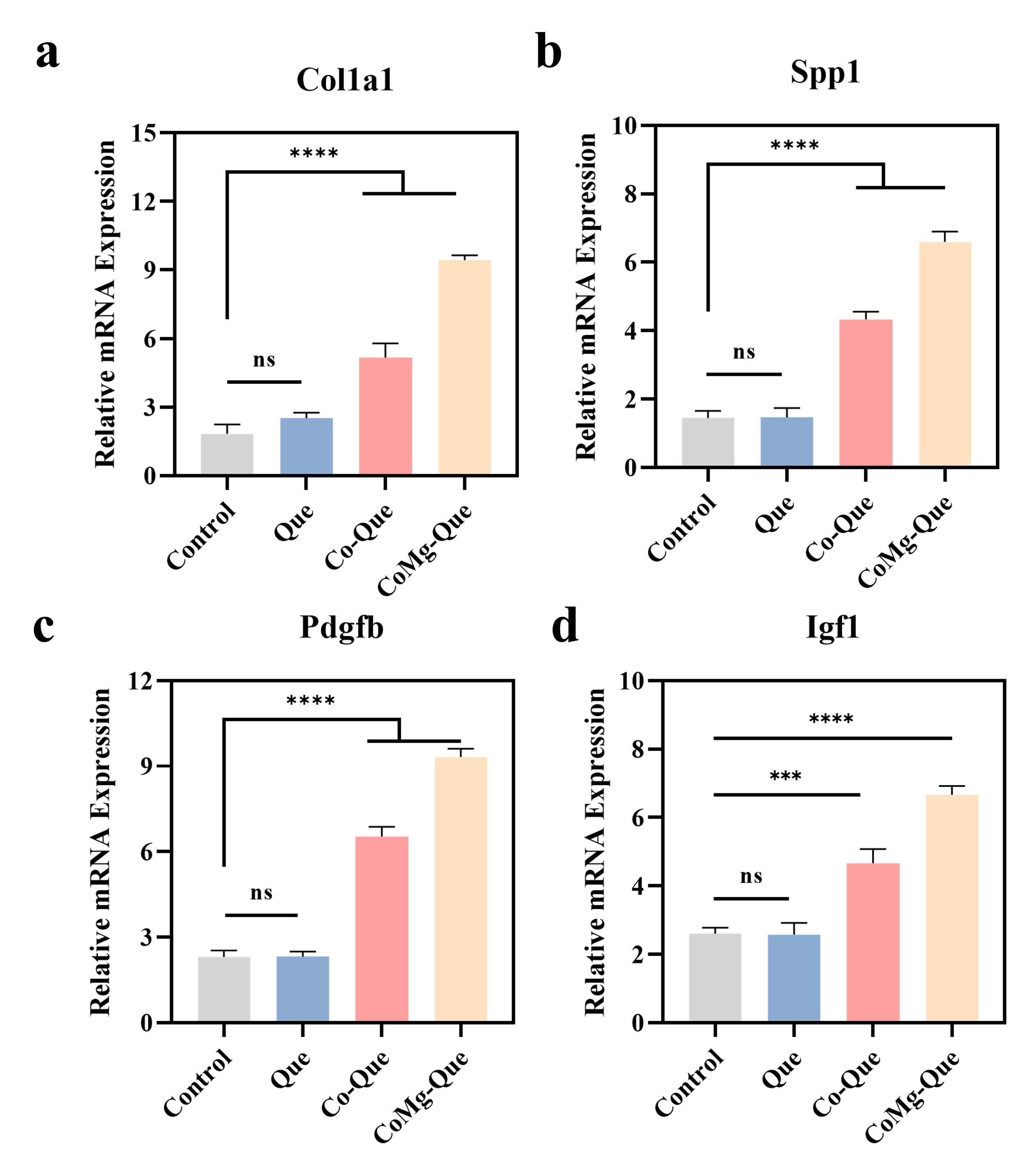
**

**Fig. S16 Relative mRNA expression levels of (a) Col1a1, (b) Spp1, (c) Pdgfb, and (d) Igf1 in BMSCs treated with different materials at day 14.** **All data are presented as mean ± SD (n = 3). ns represents p > 0.05, * represents p < 0.05, ** represents p < 0.01, *** represents p < 0.001, and **** represents p < 0.0001.**

**
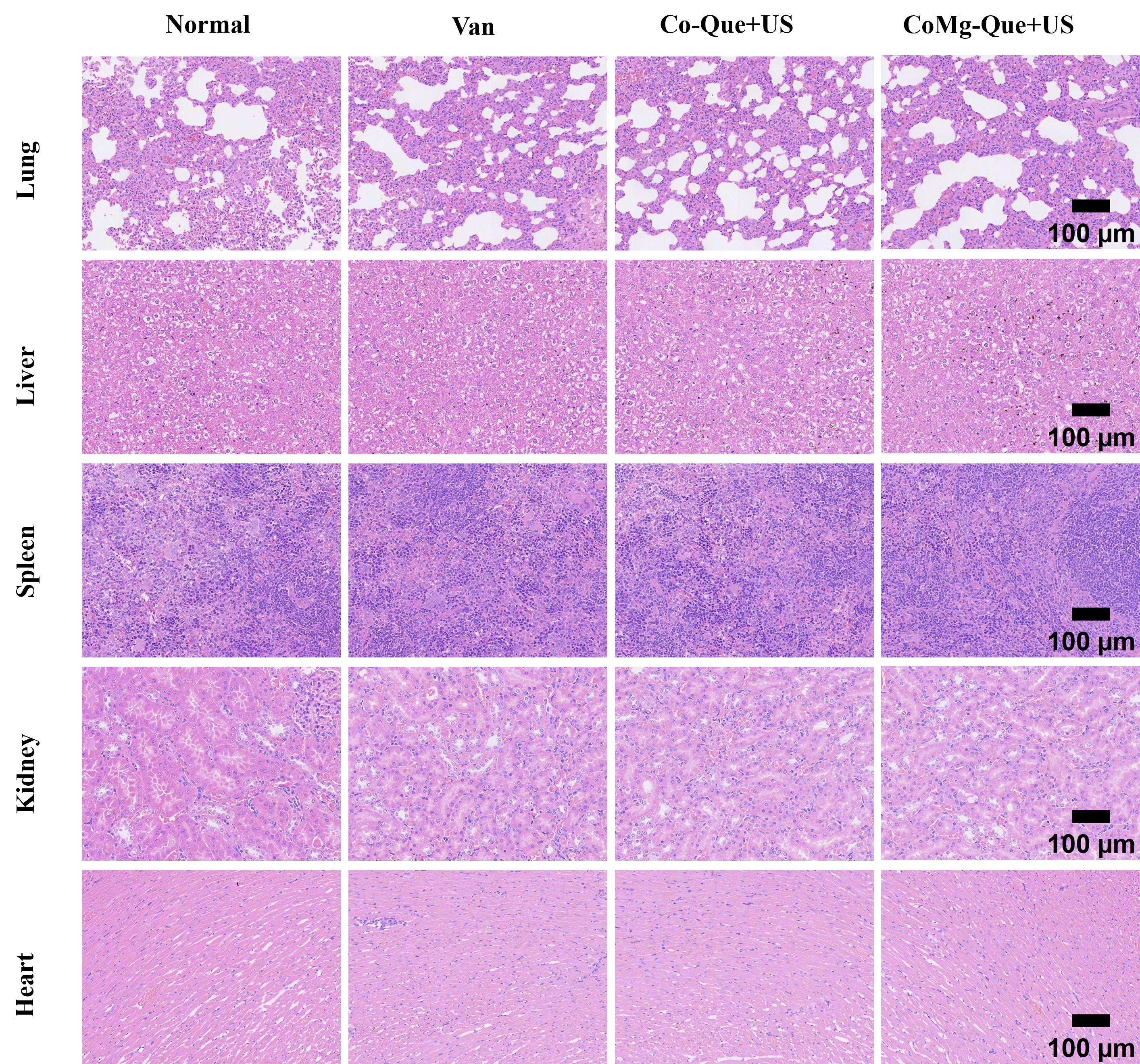
**

**Fig. S17 H&E staining of major organ tissues. (n = 3)**
